# Piplartine eliminates CD34 + AML stem/progenitor cells by inducing oxidative stress and suppressing NF-κB signalling

**DOI:** 10.1038/s41420-024-01909-4

**Published:** 2024-03-19

**Authors:** Ana Carolina B. da C. Rodrigues, Suellen L. R. Silva, Ingrid R. S. B. Dias, Rafaela G. A. Costa, Maiara de S. Oliveira, Milena B. P. Soares, Rosane B. Dias, Ludmila F. Valverde, Clarissa A. G. Rocha, Emily M. Johnson, Cristina Pina, Daniel P. Bezerra

**Affiliations:** 1 Gonçalo Moniz Institute, Oswaldo Cruz Foundation (IGM-FIOCRUZ/BA), Salvador, Bahia 40296-710 Brazil; 2 College of Health, Medicine and Life Sciences, Brunel University London, Uxbridge, UB8 3PH UK; 3 SENAI Institute for Innovation in Advanced Health Systems, SENAI CIMATEC, Salvador, Bahia 41650-010 Brazil; 4 Department of Propaedeutics and Integrated Clinical, Faculty of Dentistry, Federal University of Bahia (UFBA), Salvador, Bahia 40301-155 Brazil; 5 Center for Biotechnology and Cell Therapy, D’Or Institute for Research and Education (IDOR), Salvador, Bahia 41253-190 Brazil; 6 Centre for Genome Engineering and Maintenance, Brunel University London, Uxbridge, UB8 3PH UK

**Keywords:** Acute myeloid leukaemia, Cancer stem cells, Pharmacology

## Abstract

Acute myeloid leukaemia (AML) is a haematological malignancy characterised by the accumulation of transformed myeloid progenitors in the bone marrow. Piplartine (PL), also known as piperlongumine, is a pro-oxidant small molecule extracted from peppers that has demonstrated antineoplastic potential in solid tumours and other haematological malignancies. In this work, we explored the potential of PL to treat AML through the use of a combination of cellular and molecular analyses of primary and cultured leukaemia cells in vitro and in vivo. We showed that PL exhibits in vitro cytotoxicity against AML cells, including CD34^+^ leukaemia-propagating cells, but not healthy haematopoietic progenitors, suggesting anti-leukaemia selectivity. Mechanistically, PL treatment increased reactive oxygen species (ROS) levels and induced ROS-mediated apoptosis in AML cells, which could be prevented by treatment with the antioxidant scavenger *N*-acetyl-cysteine and the pancaspase inhibitor Z-VAD(OMe)-FMK. PL treatment reduced *NFKB1* gene transcription and the level of NF-κB p65 (pS536), which was depleted from the nucleus of AML cells, indicating suppression of NF-κB p65 signalling. Significantly, PL suppressed AML development in a mouse xenograft model, and its combination with current AML treatments (cytarabine, daunorubicin and azacytidine) had synergistic effects, indicating translational therapeutic potential. Taken together, these data position PL as a novel anti-AML candidate drug that can target leukaemia stem/progenitors and is amenable to combinatorial therapeutic strategies.

## Introduction

Acute myeloid leukaemia (AML) comprises a heterogeneous group of diseases with unique molecular and clinical characteristics. AML is the most common type of acute leukaemia, and its incidence increases with age, with most patients being older than 70 years [1–4]. In the United States of America, the estimates for 2023 were ~20,380 new cases of AML and ~11,310 deaths, with a 5-year relative survival rate of only 28% in patients aged >20 years. Under age 20, the 5-year relative survival rate is 69% [5].

The incidence of AML is increasing worldwide; however, the standard therapy has remained nearly unchanged for decades, and there have been few advances, particularly for older AML patients [6, 7]. Current standard cytotoxic regimens that involve the combination of cytarabine (ARA-C) and anthracycline (e.g., doxorubicin, DOX, daunorubicin, DNR, or idarubicin, IDA) have high toxicity, including cardiac side effects, and are not tolerated by most elderly patients [8, 9]. Newer approved treatments such as azacytidine (AZA) and venetoclax have low remission and high relapse rates, particularly in elderly individuals, altogether contributing to poor prognosis [10].

AML progression is sustained by leukaemia-propagating cells with stem-like characteristics (AML-LSCs), which need to be eliminated to achieve clinical remission and are the cause of relapse. Among the subsets of AML subtypes characterised by the expression of the CD34 stem/progenitor marker, AML-LSCs exhibit a CD34^+^CD38^-^ phenotype and can express other markers, including CD123, CD45RA, CD96, CLL-1, TIM-3, CD93 and CD99 [3, 4, 11, 12]. The elimination of AML-LSCs is central to achieving long-term remission and preventing relapse and should be specifically monitored when testing new drugs.

**Fig. 1:**
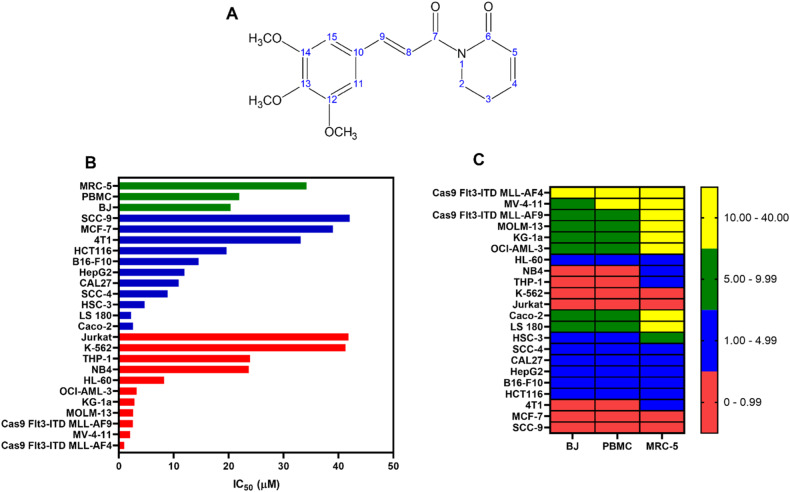
PL induced cytotoxicity against haematological and solid cancers. **A** Chemical structure of PL. **B** IC_50_ values indicating the cytotoxicity of PL against haematological (red bars) and solid cancers (blue bars), as well as against noncancerous cells (green bars). **C** Heatmap of selectivity indexes (SI) obtained for PL. The SI was calculated using the formula IS = IC_50_ [noncancerous cells]/IC_50_ [cancer cells].

Piplartine (PL, Fig. 1A), also known as piperlongumine, is a plant-based pro-oxidant small molecule with antineoplastic potential [13–18]. It has shown promising results in suppressing the growth and proliferation of malignant blood cells; for example, it has been used to treat multiple myeloma [19], lymphoma [20] and chronic myelogenous leukaemia [21]. CD34^+^ AML cells with acquired aberrant glutathione metabolism are sensitive to PL [22]. Interestingly, the induction of oxidative stress and the inhibition of specific signalling pathways, including the NF-κB pathway, have been reported as molecular targets for PL [15, 23, 24].

In this work, we explored the anti-AML potential of PL in vitro and in vivo. We found that PL, which do not extend to healthy haematopoietic progenitors, exhibits potent cytotoxicity in leukaemia cells. PL reduces CD34^+^ LSCs by inducing oxidative stress and suppressing NF-κB signalling and has synergistic effects on ARA-C, suggesting translational value.

## Results

### PL has potent cytotoxic effects on leukaemia cells, sparing healthy haematopoietic progenitor activity

Our initial focus was to explore the cytotoxic effects of PL on different types of cancer and noncancerous cells. We started by treating a panel of 28 cell lines representing 11 tumour types, including solid and haematological malignancies. The cells were treated at serial concentrations for 72 h, after which cell viability was determined by cell counting with trypan blue or through colorimetric viability assays with MTT and Alamar blue (Fig. 1B, Fig. S1).

The IC_50_ values for haematological cancers ranged from 0.96 µM for the Cas9 Flt3-ITD MLL-AF4 AML cell line to 41.83 µM for the Jurkat T-acute lymphoblastic leukaemia cell line; for solid cancers, they ranged from 2.57 µM for the Caco-2 colon cancer cell line to 42.04 µM for the SCC-9 oral squamous cell cancer cell line (Table S1). Treatment with different concentrations of PL markedly decreased the viability of both haematological and solid tumour cells. Interestingly, AML cells harbouring KMT2A rearrangements in vitro transformed mouse bone marrow (BM), Cas9 *Flt3-ITD* MLL-AF4 (IC_50_ = 0.96) and Cas9 *Flt3-ITD* MLL-AF9 (IC_50_ = 2.55), and human MV-4-11 (IC_50_ = 2.05) and MOLM-13 (IC_50_ = 2.58), which have a mutational spectrum equivalent to that of transformed mouse BM lines, were especially sensitive to PL.

PL was also evaluated for its cytotoxicity in noncancerous cell lines, including MRC-5 (derived from normal human lung tissue) and BJ (a human fibroblast line), as well as in proliferating peripheral blood mononuclear cells (PBMCs). The IC_50_ values obtained were 34.19, 21.91 and 20.34 μM for MRC-5 cells, PBMCs, and BJ cells, respectively. These noncancerous cells were included in the study to assess the selectivity and potential toxicity of PL toward normal cells, providing valuable insights into its therapeutic window and safety profile. Figure 1C shows the heatmap of the obtained selectivity indexes. DOX was used as a positive control and had IC_50_ values ranging from 0.01 µM for the Jurkat cell line to 2.1 µM for the SCC-4 cell line and IC_50_ values of 1.65, 1.21 and 3.60 μM for the noncancerous MRC-5, PBMC and BJ cells, respectively.

Cell viability was also determined after 12, 24, 48 and 72 h of incubation with PL using the trypan blue exclusion assay in the CD34^+^ phenotype AML cell line KG-1a. PL reduced the viability of KG-1a cells in a time- and concentration-dependent manner (Fig. S2). After 12 h and 24 h of treatment, the reductions were 47.4% and 59.9% (5 µM), 49.7% and 62.7% (10 µM) and 65.6% and 64.4% (20 µM), respectively. A reduction of 71.6% and 93.9%, 74.1% and 94.1% and 76.0% and 96.0% was observed after 48 and 72 h of treatment at the same concentrations, respectively.

**Fig. 2:**
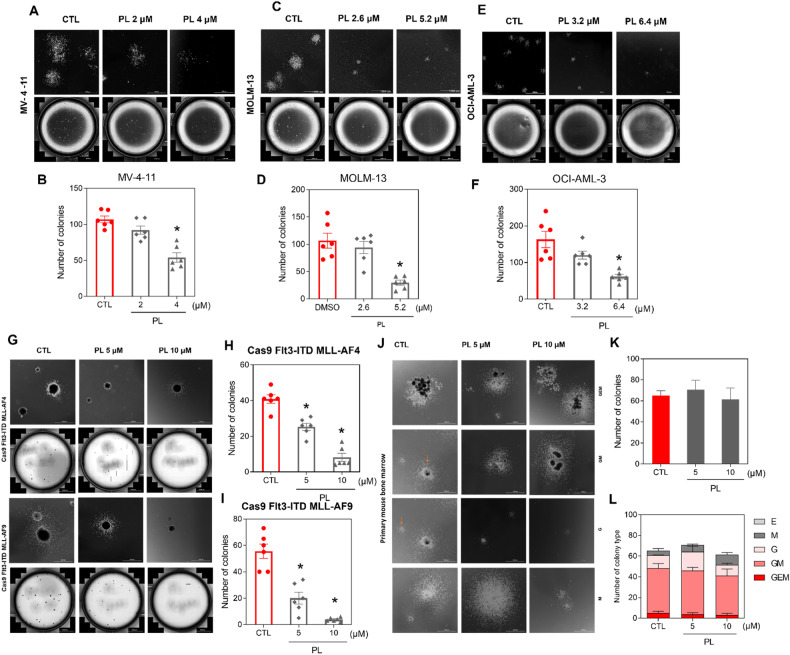
Effects of PL on the colony formation of human and mouse AML cell lines and normal primary bone marrow cells. Colony-forming ability of (**A**, **B**) MV4-11, (**C**, **D**) MOLM-13, (**E**, **F**) OCI-AML-3 and (**G**, **H**) Cas9 FLT3-ITD MLL-AF4 and (**G**, **I**) MLL-AF9 cells after treatment with PL at different concentrations and representative colony formation microscopy images. **J** Representative colony formation microscopy images of primary mouse bone marrow cells. **K**, **L** Effects of PL on the total number of colonies and the number of different colony types derived from primary mouse bone marrow cells. Colony-forming cell: M, monocyte; G, granulocyte; GM, granulocyte/macrophage; GEM, granulocyte, erythrocyte, and macrophage. The colony type is indicated by red arrows. The vehicle (0.2% DMSO) was used as a negative control (CTL). Scale bar: 1000 μm. The data are shown as the mean ± S.E.M. of three independent experiments carried out in duplicate. * *p* < 0.05 compared to CTL by one-way ANOVA followed by Dunnett’s multiple comparisons test.

Since AML models with KMT2A rearrangements are particularly sensitive to PL treatment, we performed colony-forming cell (CFC) assays to determine whether PL affects the ability of AML cell lines harbouring KMT2A rearrangements (MV-4-11, MOLM-13, Cas9 Flt3-ITD MLL-AF4 and MLL-AF9) to initiate colony formation, an in vitro surrogate for leukaemia stem/progenitor activity. In addition, we tested the effects of PL on colony formation in OCI-AML-3 cells, an AML cell line with *DNMT3A* and *NPM1* mutations, which, similar to KMT2A rearrangements, also activate a *HOX* oncogenic programme. As shown in Fig. 2A–F, PL treatment reduced the ability of human AML MV-4-11, MOLM-13 and OCI-AML-3 cells to form colonies. We also observed a significant reduction in the number of CFCs in transformed mouse BM Cas9 Flt3-ITD MLL-AF4 and MLL-AF9 cells after treatment with 5 or 10 μM (Fig. 2G–I). These data indicate that PL cytotoxicity extends to colony-forming cells, indicating its potential impact on leukaemia-propagating progenitors, particularly in vitro.

In contrast, the colony-forming potential of normal primary bone marrow cells from C57BL/6 mice was not affected by PL treatment at 5 or 10 μM. Mouse CFCs are classified according to their progenitor origin as follows: GEM (granulocyte, erythroid and monocyte); GM (granulocyte-monocyte); G (granulocyte); M (monocyte); and E (erythroid) colonies. The effects of each type of PL were quantified to assess the potential toxic effects of PL on the differentiation or proliferation of different healthy progenitors. As shown in Fig. 2J–L, compared with the control treatment, PL treatment did not significantly affect any of the colony types.

### PL targets candidate LSCs and suppresses AML development in a mouse xenograft model

**Fig. 3:**
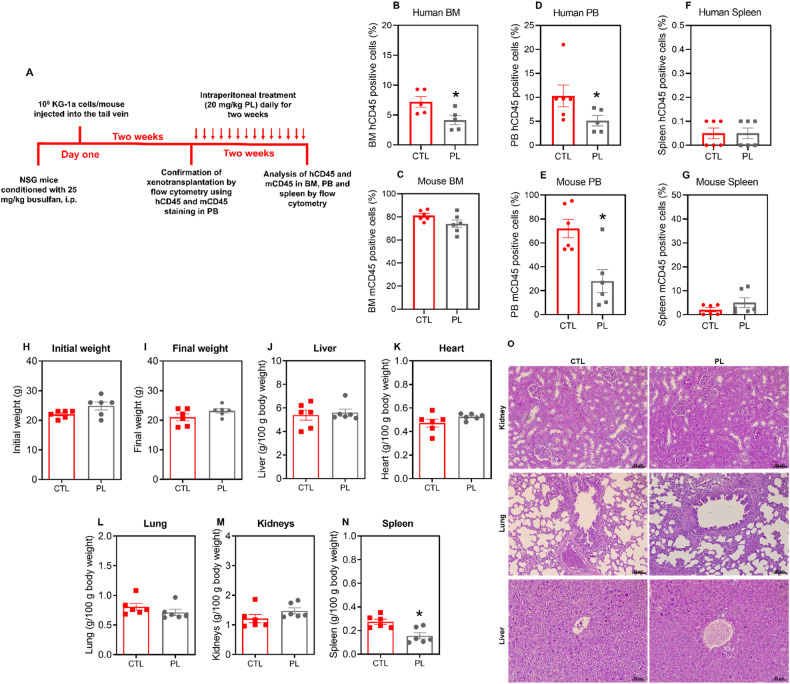
Effect of PL on the growth of xenografts derived from KG-1a cells. **A** A xenograft model was established in NSG mice. Two weeks after the inoculation of KG-1a cells, the mice were randomly divided into a PL (20 mg/kg) group and a control group (5% DMSO). hCD45-positive cells were quantified by flow cytometry from the (**B**) bone marrow (BM), (**D**) peripheral blood (PB) and (**F**) spleen. mCD45-positive cells in the (**C**) BM, (**E**) PB and (**G**) spleen were quantified via flow cytometry. All animals were weighed at the beginning (**H**) and at the end (**I**) of the experiment. The relative weights of the liver (**J**), heart (**K**), lung (**L**), kidney (**M**) and spleen (**N**) were measured at the end of the experiment. **O** Representative photomicrographs of kidneys, lungs and livers (scale bar = 50 µm). Kidneys exhibited mild glomerular hyalinisation, decreased urinary space, vascular hyperaemia, focal areas of coagulation necrosis, inflammation and fibrosis. The lungs showed thickened alveolar septa, atelectasis, hyperaemia, inflammation, oedema, haemorrhage, fibrosis, and focal areas of haemosiderin deposition. The liver displayed vascular hyperaemia, tissue inflammation, hydropic degeneration, and hepatocyte coagulation necrosis. However, no significant differences were observed between these two groups, except for certain areas of coagulative necrosis, which were more pronounced in the PL-treated mice. These alterations are reversible, except for specific necrotic areas in hepatocytes and renal tubules. The data are shown as the mean ± S.E.M. of 5–6 animals. *p < 0.05 compared with CTL according to Student’s *t* test.

Having demonstrated the specific effect of PL on colony-forming leukaemia progenitors in vitro, we tested whether this effect extended to an in vivo xenotransplantation setting. For this purpose, we focused on the KG-1a AML cell line, which displays a CD34^+^ phenotype resembling the more clearly defined human AML-LSCs [25]. For this purpose, KG-1a cells were grafted into mice to establish a leukaemia model (Fig. 3A). As shown in Fig. 3B–G, PL treatment significantly inhibited leukaemia growth in vivo. The number of engrafted human CD45^+^ cells in the bone marrow (BM) and peripheral blood (PB) was significantly lower in the mice treated with PL than in the negative control group. Mouse CD45-positive cells also showed a reduction in the PB; however, there was no statistically significant difference in the spleen or BM, suggesting selectivity against the leukaemia graft.

In support of the low toxicity to normal tissues, no significant change in total body weight or in individual organ weight was observed, except for the spleen (Fig. 3H–N). Histopathological analysis of the kidneys, heart, lungs and liver of PL-treated and control animals revealed similar alterations in both groups, most of which were mild and/or reversible (Fig. 3O). Importantly, we did not observe pathological changes in the heart, a critical toxicity target of conventional anti-AML therapies.

**Fig. 4:**
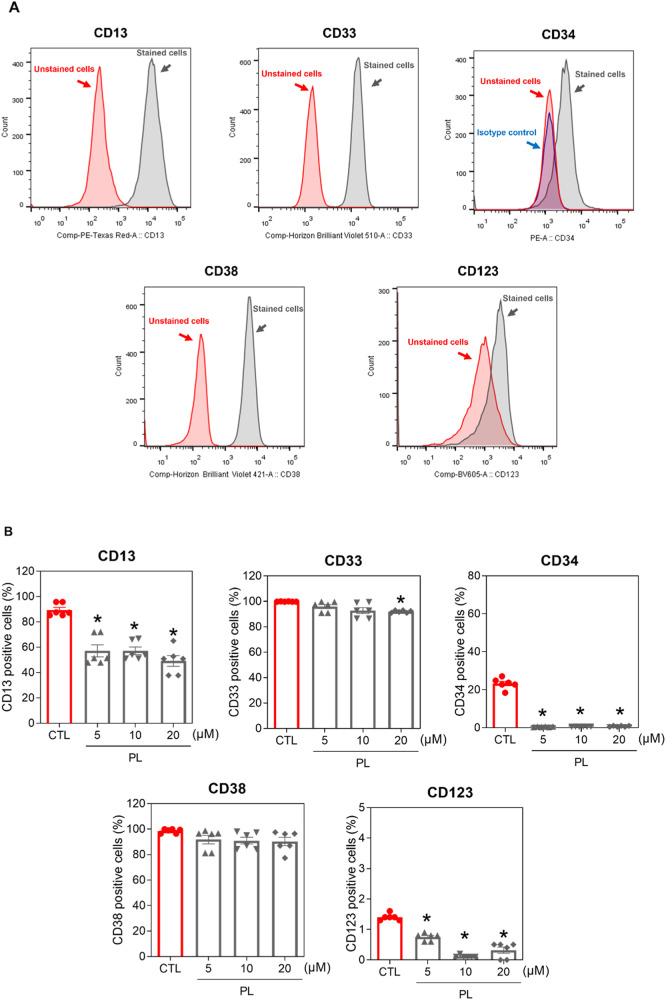
PL treatment reduced AML stem/progenitor cells into KG-1a cells. **A** Representative histograms of CD13, CD33, CD34, CD38 and CD123 expression in KG-1a cells. PE mouse anti-human CD34, BV421 mouse anti-human CD38, BV605 mouse anti-human CD123, PE-CF594 mouse anti-human CD13 and BV510 mouse anti-human CD33 antibodies were used. PE mouse IgG1, a κ isotype control, was used as an isotype control, and annexin V-FITC or YO-PRO-1 was used to select viable cells. **B** Effect of PL treatment on the antigen expression level of AML stem/progenitor cell markers in KG-1a cells. Flow cytometry was used to detect the antigen expression of CD13, CD33, CD34, CD38 and CD123. The vehicle (0.2% DMSO) was used as a negative control (CTL). The data are shown as the mean ± S.E.M. of three independent experiments carried out in duplicate. *p < 0.05 compared to CTL by one-way ANOVA followed by Dunnett’s multiple comparisons test.

In addition to its effect on LSC drug efficacy, phenotypic analysis of KG-1a AML cells treated with PL for 48 h in vitro revealed loss of the AML stem/progenitor markers CD34 and CD123, as well as the myeloid marker CD13, at all concentrations tested (5, 10 and 20 μM) (Fig. 4). The early myeloid marker CD33 was significantly reduced at a concentration of 20 μM. On the other hand, no changes were observed in CD38, which is a marker of proliferative progenitors but not LSCs (Fig. 4).

### PL induces caspase-mediated apoptotic cell death in KG-1a AML cells

**Fig. 5:**
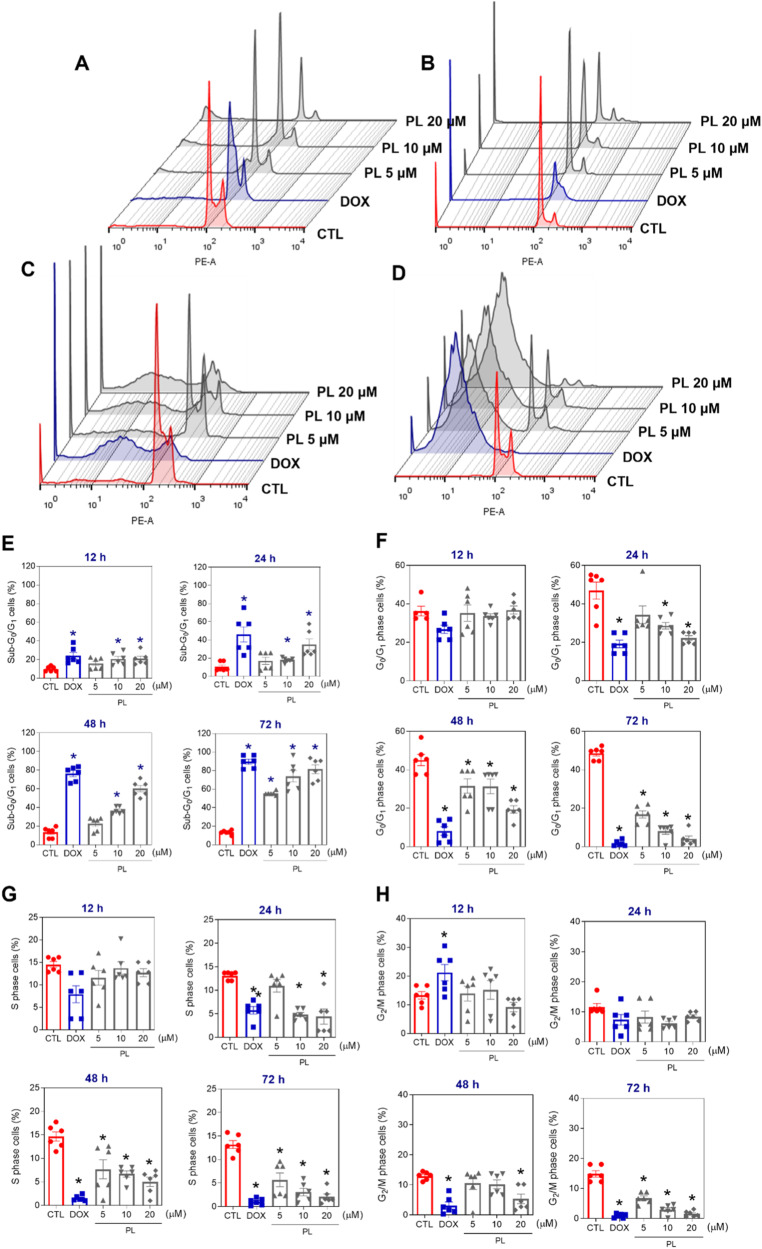
DNA fragmentation and cell cycle progression in KG-1a cells after treatment with PL. Representative histograms after (**A**) 12, (**B**) 24, (**C**) 48 and (**D**) 72 h of treatment. Percentages of cells in (**E**) sub-G_0_/G_1_, (**F**) G_0_/G_1_, (**G**) S and (**H**) G_2_/M after different incubation periods with PL. Vehicle (0.2% DMSO) was used as a negative control (CTL), and doxorubicin (DOX, 1 µM) was used as a positive control. The data are the mean ± S.E.M. of three independent experiments in duplicate. *p < 0.05 compared with CTL by one-way ANOVA followed by Dunnett’s multiple comparisons test.

To elucidate the mechanism underlying the cytotoxicity associated with PL treatment, we investigated the induction of apoptosis and tested caspase activation. First, flow cytometry was used to measure the DNA content to quantify internucleosomal DNA fragmentation and cell cycle progression in KG-1a cells after treatment with PL (Fig. 5A-H). PL treatment was capable of inducing DNA fragmentation (*p* < 0.05). After 48 h of treatment with PL, the highest concentration (20 μM) of PL significantly increased the percentage (60.2%) of the sub-G_0_/G_1_ fraction compared to that of the control group. At 5, 10 and 20 μM, DNA fragmentation significantly increased by 55.4%, 73.9% and 81.5%, respectively, after 72 h of treatment. DNA fragmentation is a central feature of apoptosis and can be used as a marker of apoptotic cell death. Doxorubicin also induced DNA fragmentation after 12, 24, 48 and 72 h.

**Fig. 6:**
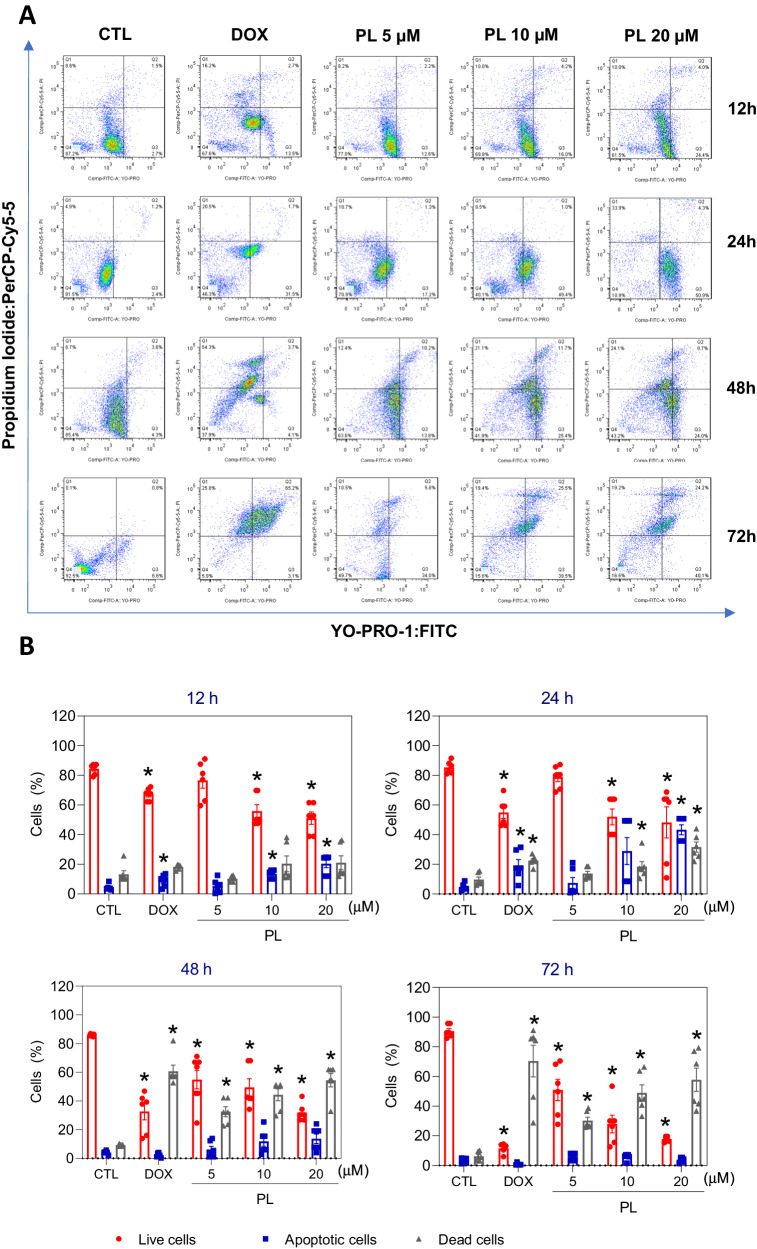
Apoptotic cell death induced by PL in KG-1a cells. **A** Representative flow cytometry dot plots. **B** Quantification of apoptosis in KG-1a cells after 12, 24, 48 and 72 h of treatment with PL. Quantification of live (YO-PRO-1- and PI-negative cells), apoptotic (YO-PRO-1-positive cells) and dead (YO-PRO-1- and PI-positive cells) KG-1a cells. Vehicle (0.2% DMSO) was used as a negative control (CTL) and doxorubicin (DOX, 1 µM) was used as a positive control. The data are shown as the mean ± S.E.M. of three independent experiments carried out in duplicate. *p < 0.05 compared with CTL by one-way ANOVA followed by Dunnett’s multiple comparisons test.

The presence of apoptotic cells was measured in PL-treated KG-1a cells using YO-PRO-1/propidium iodide (PI) double staining by flow cytometry after 12, 24, 48 and 72 h (Fig. 6A, B) for quantification of viable (YO-PRO-1/PI-double-negative cells), apoptotic (YO-PRO-1-positive cells PI-negative cells), and dead cells (YO-PRO-1/PI-double-positive cells), respectively. PL treatment at 10 and 20 μM caused a significant increase in the percentage of apoptotic cells after 12 and 24 h. After 48 and 72 h of treatment with PL, the number of dead cells increased at 5, 10 and 20 μM. The positive control, DOX (1 μM), also significantly increased the percentage of apoptotic cells after 12 and 24 h of treatment.

The cellular characteristics of size and complexity/granularity were assessed using light scattering features determined by flow cytometry in PL-treated KG-1a cells after 12, 24, 48 and 72 h (Fig. S3). In this experiment, forward light scatter (FSC) was used as a cell size parameter, and side scatter (SSC) was used as a cell complexity/granularity parameter. PL treatment caused cell shrinkage, as detected by a reduction in the FSC, which was accompanied by a gain in the SSC, likely due to nuclear condensation. Both morphological changes are related to apoptotic cells. In addition, we measured apoptosis in PL-treated MOLM-13 cells after 12 h of incubation using annexin V-APC/Hoechst 33342 double staining and flow cytometry. We observed a significant increase in the number of Annexin V-positive cells after treatment with 5 or 10 μM (Fig. S4).

**Fig. 7:**
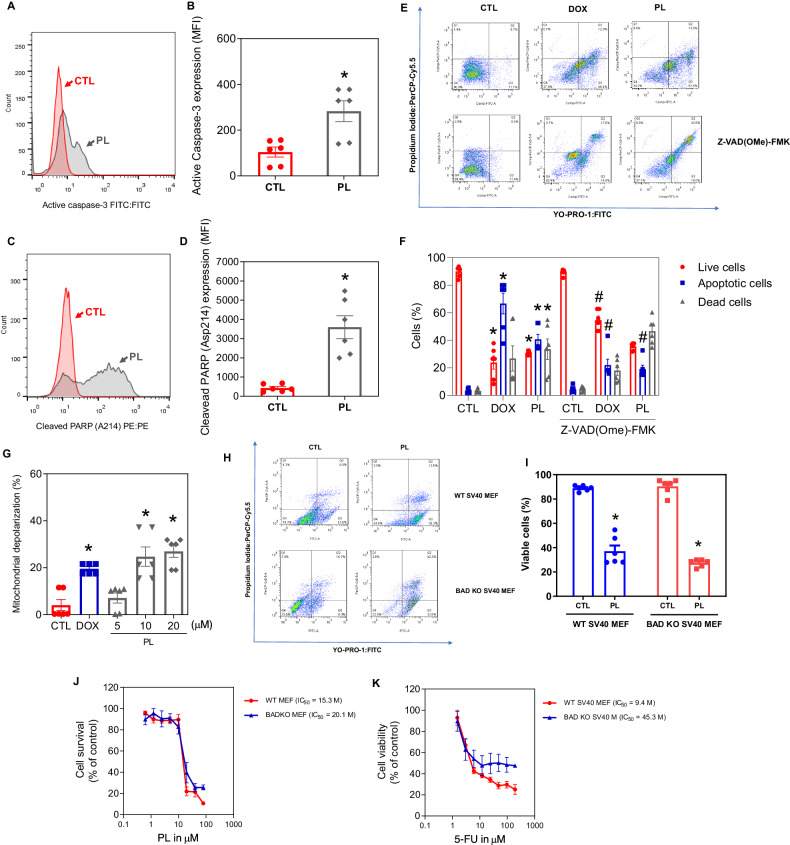
PL induced caspase-mediated apoptosis in KG-1a cells. **A,** **B** Effect of PL on the levels of active caspase 3 and (**C,** **D**) cleaved PARP (Asp214) after 24 h of treatment in KG-1a cells. Effect of the pancaspase inhibitor Z-VAD(OMe)-FMK on the apoptosis induced by PL in KG-1a cells. **E,** **F** The cells were pretreated for 2 h with 50 μM Z-VAD(OMe)-FMK and then incubated with 20 μM PL for 48 h. **G** Effect of PL on mitochondrial activity in KG-1a cells. **H,** **I** Induction of cell death in the WT SV40 MEFs and BAD KO SV40 MEFs after 48 h of incubation with 16 μM PL. **J,** **K** Survival curves of WT SV40 MEFs and BAD KO SV40 MEFs upon treatment with 5-FU and PL. The curves were obtained from at least three independent experiments carried out in duplicate using the Alamar blue assay after 72 h of incubation. Vehicle (0.2% DMSO) was used as a negative control (CTL), and doxorubicin (DOX, 1 µM) was used as a positive control. The data are shown as the mean ± S.E.M. of three independent experiments carried out in duplicate. *p < 0.05 compared with CTL by Student’s *t* test or one-way ANOVA followed by Dunnett’s multiple comparisons test. #*p* < 0.05 compared with the respective treatment without inhibitor by Student’s *t* test. MFI mean fluorescence intensity.

PL-mediated apoptosis induction was also tested by evaluating the activation of the caspase cascade, specifically through the quantification of active caspase-3 (Fig. 7A, B) and cleaved PARP (Asp214) (Fig. 7C, D), by flow cytometry. PL treatment at 20 µM significantly increased the levels of active caspase-3 and cleaved PARP (Asp214) in KG-1a cells after 24 h. Conversely, PL-induced apoptosis was prevented by treatment with the pancaspase inhibitor Z-VAD(OMe)-FMK (Fig. 7E, F).

Given the central role of mitochondria in apoptotic cell death, we also investigated the effects of PL treatment on the mitochondrial membrane potential of KG-1a cells and observed an increase in the percentage of mitochondrial depolarisation in PL-treated KG-1a cells (Fig. 7G). Consistent with the findings of mitochondrial dysfunction, downregulation of the expression of the antiapoptotic gene *BCL2* was detected (RQ = 0.11) (Table S2), suggesting that mitochondria contribute to PL-induced cell death. The expression of the proapoptotic genes *BAD* (RQ = 0.97), *BAX* (RQ = 0.70) and *BID* (RQ = 0.77) was unchanged (Table S2).

**Fig. 8:**
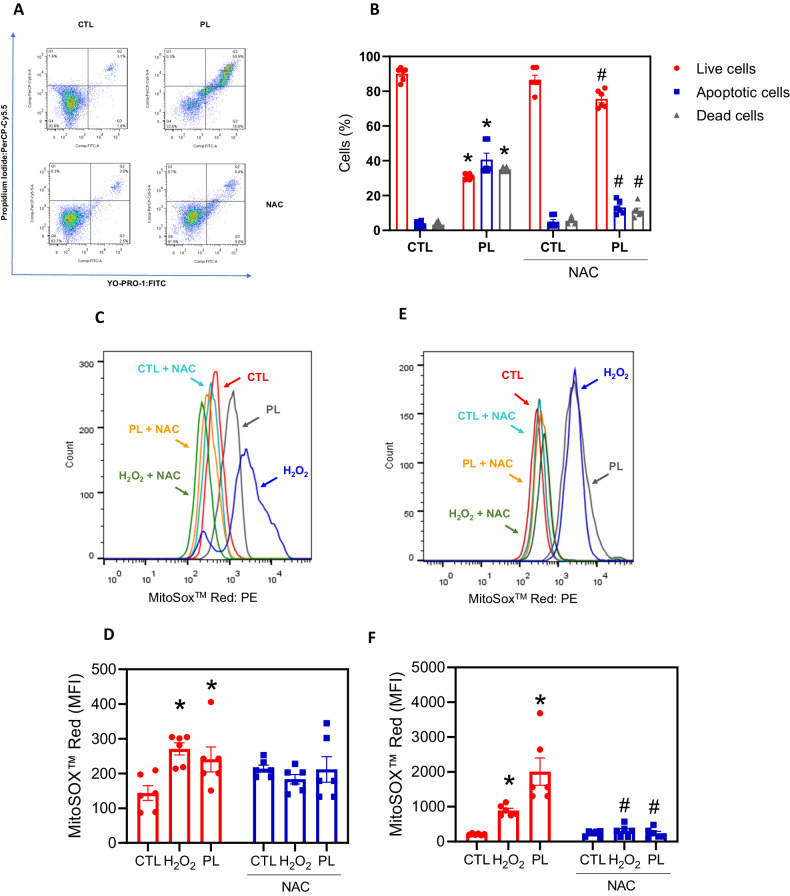
PL induced ROS accumulation in KG-1a cells. **A,** **B** Effect of the antioxidant *N*-acetyl-cysteine (NAC) on the apoptosis induced by PL in KG-1a cells. The cells were pretreated for 2 h with 5 mM NAC and then incubated with 20 μM PL for 48 h. Mitochondrial ROS in KG-1a cells after 1 (**C,** **D**) and 24 h (**E,** **F**) of treatment with PL. Vehicle (0.2% DMSO) was used as a negative control (CTL), and hydrogen peroxide (H_2_O_2_, 100 µM) was used as a positive control. The data are shown as the mean ± S.E.M. of three independent experiments carried out in duplicate. * *p* < 0.05 compared with CTL by Student’s *t* test or one-way ANOVA followed by Dunnett’s multiple comparisons test. #*p* < 0.05 compared with the respective treatment without inhibitor by Student’s *t* test. MFI mean fluorescence intensity.

Accordingly, treatment of the mouse embryonic fibroblast cell line SV40 with PL in the presence or absence of the proapoptotic gene BAD (BAD KO SV40 vs. parental WT SV40) invariably resulted in apoptosis (Fig. 7H–K), indicating that PL-driven apoptosis proceeded in a BAD-independent manner.

### PL inhibits NF-κB signalling and induces ROS-mediated apoptotic cell death in KG-1a AML cells

As mitochondria are important targets for PL-induced cell death, as these organelles play a central role in ROS production, and because PL is a known ROS inducer [14], we investigated the role of oxidative stress in PL-induced cell death in KG-1a cells. We first observed that PL-induced apoptosis was prevented by the antioxidant *N*-acetyl-cysteine (NAC) (Fig. 8A, B). We then measured mitochondrial ROS levels using the MitoSOX assay, alone or under NAC pretreatment. PL increased MitoSOX fluorescence after 1 and 24 h of treatment (Fig. 8C–F). However, MitoSOX levels decreased when KG-1a cells were pretreated with NAC (Fig. 8C–F). Taken together, these results suggest that PL induces apoptosis through increased oxidative stress.

To better elucidate the molecular mechanism underlying PL-induced apoptosis in KG-1a cells, we measured changes in the relative expression of 92 genes related to important signalling pathways in AML stem/progenitor cells. These genes included NF-κB, WNT/β-catenin, Hedgehog, NOTCH, EGFR, JAK/STAT, PI3K/Akt/mTOR, TGF-β/SMAD, and PPAR, as well as genes related to oxidative stress, apoptosis, autophagy, necroptosis and epithelial–mesenchymal transition. Gene expression analysis was performed after 12 h of incubation with PL using a qPCR TaqMan® array plate 96 plus Fast.

**Fig. 9:**
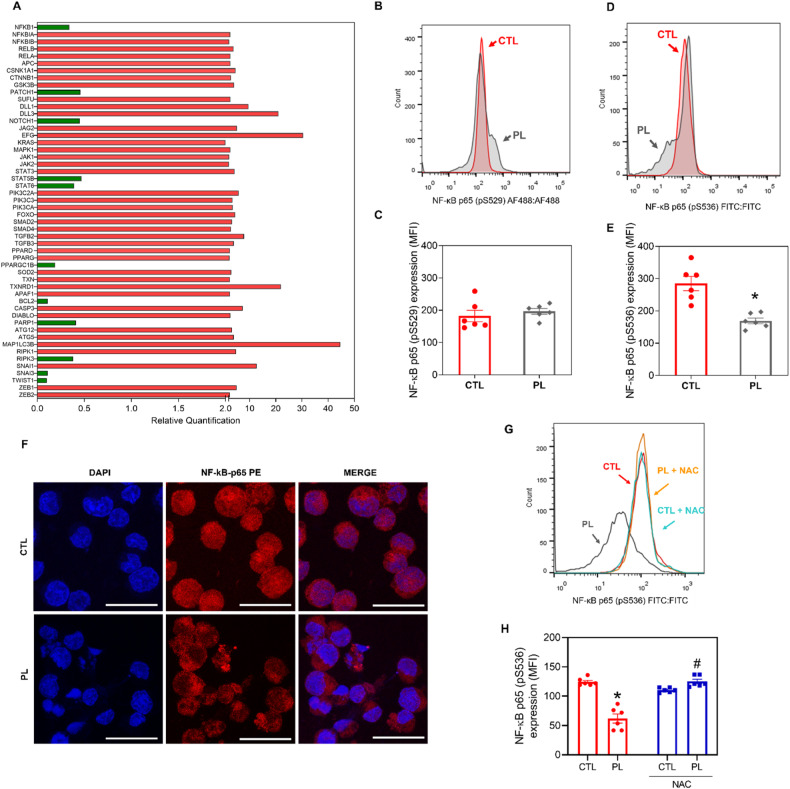
PL affected NF-κB signalling. **A** Up- and downregulated genes in KG-1a cells after 12 h of treatment with 20 µM PL. Genes that displayed RQ ≥ 2 (red bars) were upregulated, and RQ ≤ 0.5 (green bars) were downregulated. **B,** **C** Effect of PL on the levels of NF-κB p65 (pS529) after 24 h of treatment in KG-1a cells. **D,** **E** Effect of PL on the levels of NF-κB p65 (pS536) after 24 h of treatment in KG-1a cells. **F** Representative immunofluorescence images of NF-κB p65 in KG-1a cells after 24 h of incubation with 20 μM PL. Scale bar = 25 μm. **G,** **H** Effect of the antioxidant *N*-acetyl-cysteine (NAC) on the modulatory effect of PL on NF-κB p65 (pS536) levels after 24 h of treatment in KG-1a cells. The vehicle (0.2% DMSO) was used as a negative control (CTL). The data are shown as the mean ± S.E.M. of three independent experiments carried out in duplicate. * *p* < 0.05 compared with CTL according to Student’s *t* test. # *p* < 0.05 compared with the respective treatment without inhibitor by Student’s *t* test. MFI mean fluorescence intensity.

A total of 41 upregulated genes and 11 downregulated genes were identified after treatment with PL (Fig. 9A and Table S2). Among them, PL downregulated genes from the NF-κB pathway (*NFKB1)*, Hedgehog pathway (*PTCH1)*, NOTCH pathway (*NOTCH1)*, JAK/STAT pathway (*STAT5B* and *STAT6)* and PPAR pathway (*PPARGC1B)*, as well as the above mentioned antiapoptotic *BCL-2*, together with *PARP1*, necroptosis genes (*RIPK3)* and genes implicated in epithelial–mesenchymal transition (*SNAIL3* and *TWIST1)*.

We investigated the putative role of NF-κB in mediating the effects of PL on KG-1a cells by quantifying NF-κB p65 (pS536) and NF-κB p65 (pS529) protein phosphorylation levels via flow cytometry and quantifying the localisation of NF-κB p65 via confocal microscopy. Both NF-κB phosphorylation and localisation are central to its activity [26]. After 24 h of treatment with PL, although the expression of NF-κB p65 (pS529) did not change (Fig. 9B, C), the levels of NF-κB p65 (pS536) were significantly lower than those in the negative control (Fig. 9D, E). In addition, a reduction in nuclear NF-κB p65 protein was observed in PL-treated KG-1a cells (Fig. 9F), consistent with the reduction in NF-κB p65 activity. Next, we investigated whether the modulatory effects of PL on NF-κB are prevented by NAC. Curiously, the inhibitory effect of PL on NF-κB was prevented by NAC (Fig. 9G, H).

Taken together, our mechanistic analyses suggest that PL impacts NF-κB activity and ROS production, resulting in apoptosis mediated by mitochondrial regulation of oxidative stress.

### PL combined with ARA-C or DNR synergistically inhibits AML growth

Having evaluated the anti-AML activity of PL and dissected its mechanisms of action, we tested its translational potential by evaluating the value of its combination with drugs currently used in AML therapy. We specifically tested combinations of ARA-C, DNR, AZA and PL and focused on cell lines highly responsive to PL, which represent common and/or severe forms of disease. These were KMT2A-rearranged MOLM-13, Cas9 FLT3-ITD MLL-AF4 and Cas9 FLT3-ITD MLL-AF9 cells and *NPM1* and *DNMT3A* mutant OCI-AML3 cells. Based on the calculated IC_50_ of the monotherapies, we used concentrations of PL predicted to inhibit 25, 50 and 75% of the cell growth and combined them with the IC_10_ and IC_25_ doses of ARA-C and DNR and the IC_25_ and IC_50_ doses of AZA. We deliberately assayed lower concentrations of ARA-C and DNR to assess the value of the combination of these agents with PL for enabling the use of conventional chemotherapeutic agents, which are not normally tolerated by elderly AML patients. We used the algorithm CompuSyn, which calculates the combination index (CI) and plots it against the inhibitory effect [27]. The CI is a quantitative metric used to assess the interaction between two or more drugs or treatments. It is calculated based on the concentration‒response relationship of individual drugs and their combination. A CI less than 0.9 indicated a synergistic effect. A CI in the range of 0.9-1.1 suggested an additive effect. A CI greater than 1.1 indicates an inhibitory effect.

**Fig. 10:**
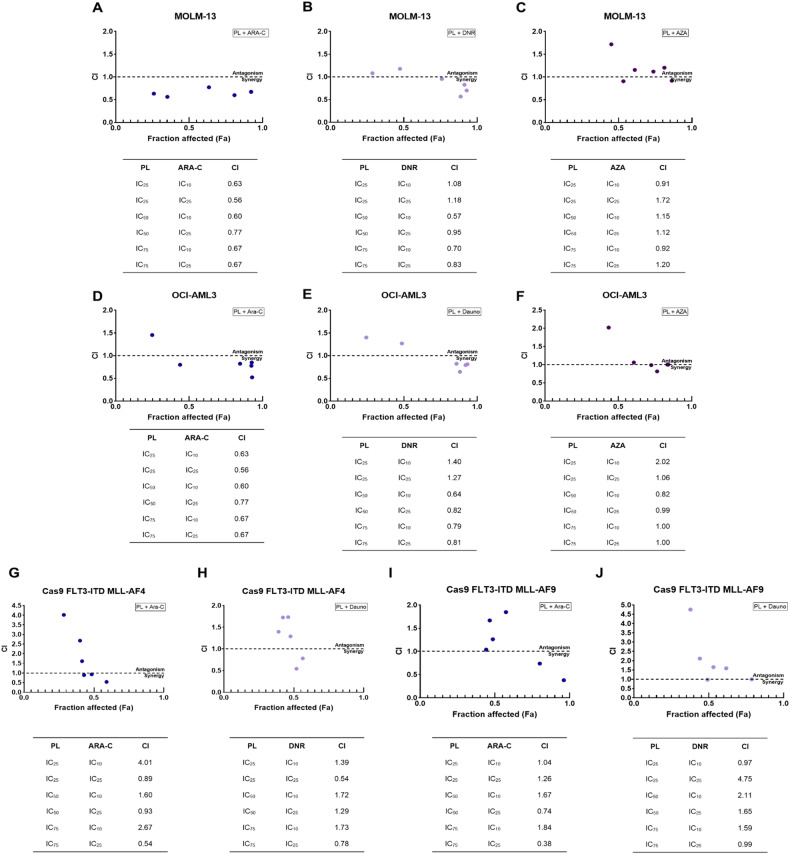
Effects of combination treatment with PL plus ARA-C, DNR or Aza on the growth of AML cells in vitro. Combination index plot (Fa-CI plot) of the interactions between (**A**) PL and ARA-C, (**B**) PL and DNR and (**C**) PL and AZA in MOLM-13 cells, (**D**) PL and ARA-C, (**E**) PL and DNR and (**F**) PL and AZA in OCI-AML3 cells, (**G**) PL and ARA-C and (**H**) PL and DNR in Cas9 FLT-ITD MLL-AF4 cells and (**I**) PL and ARA-C and (**J**) PL and DNR in Cas9 FLT-ITD MLL-AF9 cells generated by CompuSyn software. Fa: inhibitory effect, CI: combination index. AML cells were treated for 72 h with the control vehicle, individual drugs or the 2-drug combination at different concentrations on the basis of previously established IC_50_ values, after which cell proliferation was measured using the MTT assay. The data are presented as the means ± S.E.M. The synergistic, additive, and antagonistic effects of drugs were defined by CI values < 1.0, 1.0, and > 1.0, respectively.

Figure 10A–C shows the CI plot of the combinations in MOLM-13 cells. The mean CI values for PL/ARA-C and PL/DNR were 0.65 ± 0.02 and 0.88 ± 0.09, respectively, indicating a synergistic effect of combination therapy on these cells, which was clearer for the PL/ARA-C combinations. On the other hand, the mean CI value of the PL/AZA combination was 1.17 ± 0.12, indicating broad additive effects.

In the case of OCI-AML3 (Fig. 10D–F), the mean CI values for PL/ARA-C and PL/DNR were 0.87 ± 0.12 and 0.95 ± 0.12, respectively, which are suggestive of additive effects. The PL/AZA combination displayed an average CI of 1.15 ± 0.18, which was also additive.

In the mouse primary bone marrow cell lines Cas9 FLT-ITD MLL-AF4 and MLL-AF9 (Fig. 10G–J), only the highest concentrations of ARA-C (50.2 and 32.44 nM, respectively) combined with PL had synergistic effects on both cell lines (0.78 ± 0.12 and 0.79 ± 0.25, respectively). All combinations of DNR and PL in these cell lines were additive. Both cell lines were highly sensitive to AZA treatment, and it was not possible to perform combinatorial analysis.

Overall, we detected consistent synergistic effects between PL and ARA-C, which were specific to KMT2A-rearranged cell lines and may be explored in patients receiving low-ARA-C regimens. No synergistic effects were observed between PL and DNR or AZA.

## Discussion

The landscape of AML treatment has undergone limited advancement over the years. AML predominantly affects middle-aged and older patients. Despite improvements in therapy, the mortality of patients with AML has remained high and is still associated with poor survival, particularly in the elderly population [28]. New therapies are urgently needed, with a focus on more effective lower-intensity therapy for patients who are unable to tolerate intensive standard chemotherapy regimens [29].

Herein, we explored the anti-AML potential of PL and found that this molecule has potent cytotoxic effects on leukaemia cells, sparing healthy haematopoietic progenitors, an effect that agrees with prior observations of cytotoxicity against malignant blood cells [19–22]. Mechanistically, PL affects mitochondrial activity and induces oxidative stress-mediated apoptosis via the suppression of NF-κB signalling. Importantly, PL reduced the CD34 + AML stem/progenitor cell subpopulation in KG-1a cells and hindered leukaemia development in a mouse xenograft model, highlighting its potential to target LSCs. PL treatment was well tolerated by the mice, and the side effects observed were considered reversible. This finding is in agreement with previous studies reporting that PL is safe for in vivo administration [15, 16, 30].

NF-κB is constitutively active in 40% of AML patients, and its aberrant activity enables leukaemic blasts and AML-LSCs to escape apoptosis and increase cell proliferation [3, 31–33]. Specifically, a functional and comparative genomic study reported that NF-κB is critical for sustaining KMT2A rearrangements in the AML stem/progenitor cell subpopulation [34], which is compatible with the high sensitivity of *KMT2A*-rearranged cells to the effects of PL. PL treatment affects breast cancer cell proliferation by suppressing IKKβ expression [35]. IKKβ suppression is associated with the accumulation of ROS, which leads to oxidative damage, thus linking the two mechanisms of action documented for PL. Pei et al. [22] also demonstrated that PL could cause glutathione depletion in stem cells of primary human AML specimens, leading to leukaemia cell death but causing less toxicity to normal haematopoietic stem cells. These findings further underline the association between PL and oxidative stress and between the susceptibility of AML to ROS toxicity, identifying an avenue for intervention in patients with this disease. Our observation of significant and selective targeting of AML cells in a xenograft model, with limited toxicity to mouse tissues, reinforces these findings. Importantly, these findings suggest the clinical potential of PL as an anti-AML candidate that can eliminate the AML stem/progenitor cell subpopulation, increasing the likelihood of prolonged remission or cure.

We found that the combination of PL and ARA-C had synergistic effects on at least AML cells harbouring *KMT2A* rearrangements. Other groups have previously demonstrated that PL can interact synergistically with different anticancer drugs. For example, PL synergises with cisplatin and paclitaxel to inhibit the growth of human ovarian cancer cells [24]. Similarly, PL and paclitaxel have synergistic cytotoxic effects on human intestinal cancer cells [36], and PL can also synergistically enhance the anticancer effects of oxaliplatin on human colorectal cancer [37]. All of these chemical drugs, including ARA-C [38–40], cause oxidative stress. Critically, they also activate the NF-κB signalling pathway as a mechanism of chemoresistance [41–46]. In the case of ARA-C, NF-κB activation is a likely mechanism underlying the failure to eliminate AML-LSCs [31]. Conversely, the synergistic effect we observed between ARA-C and PL likely results at least in part from the capacity of PL to inhibit NF-κB signalling and suggests the putative clinical utility of PL, establishing an avenue for its translational exploitation.

In conclusion, we suggest that PL be developed as anti-AML drugs capable of targeting leukaemia-propagating cells and enhancing the activity of conventional chemotherapy by targeting resistance mechanisms and allowing the use of lower doses for improved tolerability. In the future, it will be important to systematically define the AML subtypes that can benefit from PL by testing against patient cells and patient-derived xenograft models to compare the findings with those of current associations of AZA and venetoclax and to explore possible mechanisms of PL resistance for optimal and personalised association regimens.

## Materials and methods

### PL preparation

PL was obtained commercially (Cayman Chemical, Ann Arbor, MI, USA). It was dissolved in sterile DMSO (Synth, Diadema, SP, Brazil) as a 5 mg/mL stock solution and stored at −20 °C. PL was diluted in culture medium or PBS, as indicated for individual experiments.

### Cell culture

The cell lines used in this work are specified in Table S3. The cells were cultured according to the manufacturer’s instructions or according to the ATCC animal cell culture guidelines for each specific cell line or condition, as detailed in Table S3. All cell lines were grown in flasks at 37 °C in 5% CO_2_; assays were run for 3–4 days to sustain exponential growth. A 0.25% trypsin EDTA solution (Sigma‒Aldrich Co., Saint Louis, MO, USA) was used to harvest adherent cells. All cell lines were screened for mycoplasma contamination using a mycoplasma stain kit (Sigma Aldrich Co.) to validate their use as mycoplasma-free cells.

### Cell viability/proliferation assay

Cell viability/proliferation was quantified using the Alamar blue assay (Sigma‒Aldrich Co.), MTT assay (Cayman Chemical, Ann Arbor, MI, USA), or live cell counting under a microscope using trypan blue (Fisher Scientific, UK). For the Alamar blue assay, the cells were plated in 96-well culture plates (3 × 10^5^ cells/well for suspension cells and 7 × 10^4^ cells/well for adherent cells) and kept at 37 °C in a 5% CO_2_ atmosphere. The drugs were added at specified concentrations to each well in duplicate and incubated for 72 h. DOX (purity ≥ 95%, Laboratory IMA S.A.I.C., Buenos Aires, Argentina) was used as a positive control. Four hours before the end of the incubation period (or after 24 h for PBMCs), resazurin was added to each well at a final concentration of 3 μM. The absorbance at 570 nm and 600 nm was measured using a SpectraMax 190 Microplate Reader (Molecular Devices, Sunnyvale, CA, USA).

The MTT assay was carried out using an MTT Cell Proliferation Assay Kit (Cayman Chemical) according to the manufacturer’s instructions. For trypan blue-based cell counts, cells were plated in 24-well culture plates (3 × 10^5^ cells/well for suspension cells and 7 × 10^4^ cells/well for adherent cells) and kept at 37 °C in 5% CO_2_. The drugs were added to each well in duplicate and incubated for 72 h. At the end of the incubation period, live and dead cells were counted using a haemocytometer under a light microscope.

### Colony-forming assay

The MV-4-11, MOLM-13 and OCI-AML-3 cell lines were plated at a density of 500 cells/plate in duplicate in MethoCult H4435 (STEMCELL Technologies), MethoCult H4230 (STEMCELL Technologies) or StemMACS™ HSC-CFU Media (Miltenyi Biotec). Mouse bone marrow-derived Cas9 Flt3-ITD MLL-AF4 and MLL-AF9 cells [47] were plated at a density of 500 cells/plate in duplicate in mouse methylcellulose complete media HSC007 (R&D Systems). C57BL/6 mouse bone marrow cells were plated at a density of 50,000 cells/plate in the same methylcellulose-based medium, HSC007. PL was added at different concentrations to the methylcellulose solution and dispersed by vortexing prior to the addition of the cells. PL was used at the IC_50_ concentrations, as described. Colonies were counted/scored by microscopy 6–8 days after plating.

### Xenotransplantation of leukaemia cells

A total of 12 NOD. Cg-*Prkdc*^*scid*^ *Il2rg*^*tm1Wjl*^/SzJ (NSG) mice (male and female, 20–25 g) were supplied and housed under specific pathogen-free conditions by FIOCRUZ-BA animal facilities (Salvador, Bahia, Brazil) following an experimental protocol approved by a local animal ethics committee (#16/2018). All mice were fed a standard pellet diet (with free access to food and water) and kept in an artificially lit room (12 h dark/light cycle).

To achieve a high level of human cell engraftment, busulfan was used as a conditioning agent. The mice were treated with 25 mg/kg busulfan (Sigma‒Aldrich Co.) 24 h before receiving KG-1a cells. The following day, the mice were intravenously inoculated with 10^6^ cells/mouse via the tail vein. The animals were monitored every other day for signs of weight loss or lethargy. After two weeks, flow cytometry confirmed engraftment in peripheral blood using both PE-conjugated anti-human CD45 (hCD45) and FITC-conjugated anti-mouse CD45 (mCD45) antibodies. Table S4 provides a detailed description of all the antibodies used.

After engraftment confirmation, the mice were randomly divided into two groups (*n* = 6/per group): a negative control group (vehicle–DMSO 5%) and a group treated with PL at 20 mg/kg. The treatments were injected into the mice intraperitoneally every day for two weeks. Mice were euthanised after two weeks, after which cells from the spleen, peripheral blood, and bone marrow were collected. In addition, organs such as the heart, lungs, kidneys, and liver were also collected. The cells collected from the spleen, peripheral blood, and bone marrow were analysed via flow cytometry using hCD45 and mCD45 antibodies.

The heart, lungs, kidneys and liver were collected for toxicological analyses. These organs were examined for colour change, gross lesion formation, and/or haemorrhaging before being fixed in 4% formaldehyde, dehydrated in a graded alcohol series, cleaned in xylene, and embedded in paraffin wax. The tissue was cut into 5 μm thick slices, stained with haematoxylin-eosin and/or periodic acid-Schiff (liver and kidney), and examined histologically under optical microscopy by an experienced pathologist following international guidelines.

### Flow cytometry assays

Cell cycle progression was quantified based on the measurement of DNA content by staining with PI (Sigma‒Aldrich Co.) [48]. Briefly, the cells were diluted with a permeabilization solution containing 0.1% Triton X-100, 2 μg/mL PI, 0.1% sodium citrate, and 100 μg/mL RNAse (all from Sigma‒Aldrich Co.) and incubated for 15 minutes in the dark.

Apoptosis was detected and quantified using an annexin V-FITC/PI (FITC Annexin V Apoptosis Detection Kit I, BD Biosciences), an annexin V-APC/Hoechst 33258 (Biolegend/Thermo Fisher) or YO-PRO-1/PI (Sigma‒Aldrich Co.). Briefly, cells were stained with 200 μL of Annexin-V buffer (0.01 mol/L HEPES/NaOH, 0.14 mol/L NaCl, 2.5 mmol/L CaCl_2_) according to the manufacturers’ instructions; cells were washed and resuspended in staining buffer containing 1 µM Hoechst 33258 or 1.5 µM PI (incubated at room temperature for 15 min). Alternatively, the cells were stained with a solution containing 0.1 µM YO-PRO-1 and 1.5 µM PI. To examine the involvement of caspases in PL-induced apoptosis, the pancaspase inhibitor Z-VAD(OMe)-FMK (50 µM) was added to the cells 1 h prior to PL treatment.

The rhodamine 123 incorporation method was used to determine the mitochondrial transmembrane potential [49]. After 24 h of treatment, the cells were diluted in a solution of 1 μg/mL rhodamine (Sigma‒Aldrich Co.) and incubated at 37 °C in the dark for 15 min. After this period, the cells were centrifuged and resuspended in PBS.

To detect mitochondrial ROS levels, we used MitoSOX™ Red reagent (Thermo Fisher Scientific, Waltham, MA, USA), and the analysis was performed according to the manufacturer’s instructions. The cells were treated with PL and incubated for 1 or 24 h. Subsequently, the cells were washed and resuspended in a solution containing 5 µM MitoSOX™ Red. Upon entering the mitochondria, the reagent undergoes oxidation by superoxide and emits red fluorescence.

The apoptotic response to oxidative stress was determined upon treatment with the antioxidant NAC. Thus, the cells were preincubated for 2 h with 5 mM NAC, and then exposed to 20 µM PL for 48 h to evaluate the antioxidant’s protective effect against oxidative stress-induced apoptosis.

Phenotyping was carried out using antibodies against CD34, CD38, CD123, CD13 and CD33. The cells were washed with incubation buffer (0.5% bovine serum albumin in PBS), stained with PE-conjugated mouse anti-human CD34, BV421-conjugated mouse anti-human CD38, BV605-conjugated mouse anti-human CD123, PE-CF594-conjugated mouse anti-human CD13 and BV510-conjugated mouse anti-human CD33 antibodies and incubated for 1 h at room temperature. The PE mouse IgG1 κ isotype control was used as the appropriate isotype control in the present study, and apoptotic and dead cells were excluded through staining with annexin-V or YO-PRO-1. The gating strategy is detailed in Fig. S5.

For cleaved PARP (Asp 214), active caspase-3, and NF-κB p65 (pS529 and pS536) detection, cells were collected and resuspended in 0.5-1 ml of 4% formaldehyde for 10 min at 37 °C. The tubes were then placed on ice for 1 min. The cells were permeabilized for 30 min on ice by slowly adding ice-cold 100% methanol to prechilled cells while gently vortexing to a final concentration of 90% methanol. After the samples were washed with incubation buffer (0.5% bovine serum albumin in PBS), FITC-conjugated rabbit anti-active caspase-3, PE-conjugated mouse anti-cleaved PARP (Asp214), Alexa Fluor® 488-conjugated mouse anti-NF-κB p65 (pS529) or FITC-conjugated recombinant rabbit anti-NF-κB p65 (pS536) antibodies were added, and the samples were incubated at room temperature for 1 h. Finally, the cells were washed with PBS, and the fluorescence of the cells was measured using flow cytometry. DMSO (0.2%) was used as the negative control in all the experiments. Table S4 provides a detailed description of all the antibodies used in the flow cytometry experiments.

A minimum of 30,000 events/sample for cell surface staining and 10,000 events/sample for intracellular staining were analysed using a BD LSRFortessa cytometer along with BD FACSDiva Software (BD Biosciences, San Jose, CA, USA) and FlowJo Software 10 (FlowJo LCC, Ashland, OR, USA). The experiments were performed in duplicate in three independent experiments. Cell doublets and debris were excluded through gating.

### Gene expression analysis by qPCR array

KG-1a cells were incubated with 20 µM PL for 12 h, and total RNA was isolated using an RNeasy Plus Mini Kit (Qiagen; Hilden, Germany) according to the manufacturer’s instructions. The RNA was analysed for purity and quantified by a NanoDrop® 1000 spectrophotometer (Thermo Fisher Scientific, Waltham, Massachusetts, USA). RNA reverse transcription was carried out using a Superscript VILO™ Kit (Invitrogen Corporation; Waltham, MA, USA).

Gene expression qPCR analysis was performed on a TaqMan® array plate 96 plus fast (#4413256; Applied Biosystems^TM^; Foster City, CA, USA) in an ABI ViiA7 system (Applied Biosystems^TM^). The PCR cycle conditions were 2 min at 50 °C, 10 min at 95 °C, 40 cycles of 15 s at 95 °C and 1 min at 60 °C. Relative quantification (RQ) of mRNA expression was calculated using the 2^−ΔΔCT^ method [50] on Gene Expression Suite™ Software (Applied Biosystems^TM^). Cells treated with 0.2% DMSO (negative control) were used as a calibrator; normalisation of the data was performed using the geometric mean RQ of three reference genes, GUSB, HPRT1 and GAPDH. All the experiments were performed under DNase/RNase-free conditions. Genes were considered upregulated if RQ ≥ 2; similarly, genes were considered downregulated if RQ ≤ 0.5.

### Detection of NF-κB translocation by confocal microscopy

For NF-κB p65 localisation, cells were plated in 24-well plates and exposed to PL for 24 h. After the incubation period, the cells were washed twice with PBS, placed as a small drop (5 μL) on a coverslip, permeabilized with Triton X-100 (0.5%), treated with RNAse (10 μg/mL), and incubated overnight with PE mouse anti-NF-κB p65 antibody. The cells were rinsed with PBS on the next day and mounted with Fluoromount-G and DAPI (Invitrogen, Thermo Fisher Scientific). DMSO (0.2%) was used as the negative control. A Leica TCS SP8 confocal microscope (Leica Microsystems, Wetzlar, HE, Germany) was used to examine the cells. Table S4 provides a detailed description of all the antibodies used.

### Combination drug analysis

For combinatorial drug treatments, the cells were treated with PL, AZA, DNR, ARA-C or the control vehicle as single agents or in combination in a 96-well plate for 72 h. The concentrations were established according to the calculated IC_50_ values for each drug. Cell viability was determined by MTT assay, as described above. We used CompuSyn software (version 1.0; ComboSyn, Paramus, NJ, USA) to evaluate potential synergistic or additive effects through the use of isobolograms and combination-index plots and determination of the drug combination index (CI) values. CI values < 0.9 indicate a synergistic effect; CI = 0.9–1.1 indicates an additive effect; CI > 1.1 reveals an antagonistic effect [27].

### Statistical analysis

The data are shown as the mean of at least three repetitions (done in duplicate) ± S.E.M. or as IC_50_ values with a 95% confidence interval. For statistical analyses, two-tailed unpaired Student’s *t* test was used to compare data between two groups, and one-way analysis of variance (ANOVA) followed by Dunnett’s multiple comparisons test was used to compare data among three or more groups; these analyses were performed with GraphPad Prism (Intuitive Software for Science; San Diego, CA, USA).

### Supplementary information

Supplementary material

## Data Availability

Data will be made available on request.
