# Supplementary material for: Piplartine eliminates CD34 + AML stem/progenitor cells by inducing oxidative stress and suppressing NF-κB signalling
Source: Cell Death Discov. 2024 Mar 19;10:147. doi: 10.1038/s41420-024-01909-4 (PMC10951277; doi:10.1038/s41420-024-01909-4)
Supplement: Supplementary file 1 — Supplementary material [file 41420_2024_1909_MOESM1_ESM.pdf]

Supplementary Material

Piplartine eliminates CD34+ AML stem/progenitor cells by inducing oxidative stress and suppressing NF-κB signalling

Ana Carolina B. da C. Rodrigues[1,2], Suellen L. R. Silva[1], Ingrid R. S. B. Dias[1], Rafaela G. A. Costa[1], Maiara de S. Oliveira[1], Milena B. P. Soares[1,3], Rosane B. Dias[1,4], Ludmila F. Valverde[1], Clarissa A. G. Rocha[1,4,5], Emily M. Johnson[2], Cristina Pina[2,6,*,†], Daniel P. Bezerra[1,*,†]

[1]Gonçalo Moniz Institute, Oswaldo Cruz Foundation (IGM-FIOCRUZ/BA), Salvador, Bahia, 40296-710, Brazil.

[2]College of Health, Medicine and Life Sciences, Brunel University London, Uxbridge, UB8 3PH, United Kingdom.

[3]SENAI Institute for Innovation in Advanced Health Systems, SENAI CIMATEC, Salvador, Bahia, 41650-010, Brazil.

[4]Department of Propaedeutics and Integrated Clinical, Faculty of Dentistry, Federal University of Bahia (UFBA), Salvador, Bahia, 40301-155, Brazil.

[5]Center for Biotechnology and Cell Therapy, D'Or Institute for Research and Education (IDOR), São Rafael Hospital, Salvador, Bahia, 41253-190, Brazil.

[6]Centre for Genome Engineering and Maintenance, Brunel University London, Uxbridge, UB8 3PH, United Kingdom.

[†] These authors contributed equally to this work.

*Corresponding authors:

D. P. Bezerra, E-mail: daniel.bezerra@fiocruz.br

Phone number: + 55 71 3176 2272.

C. Pina, E-mail: cristina.pina@brunel.ac.uk

Phone number: +44 1895 266676.

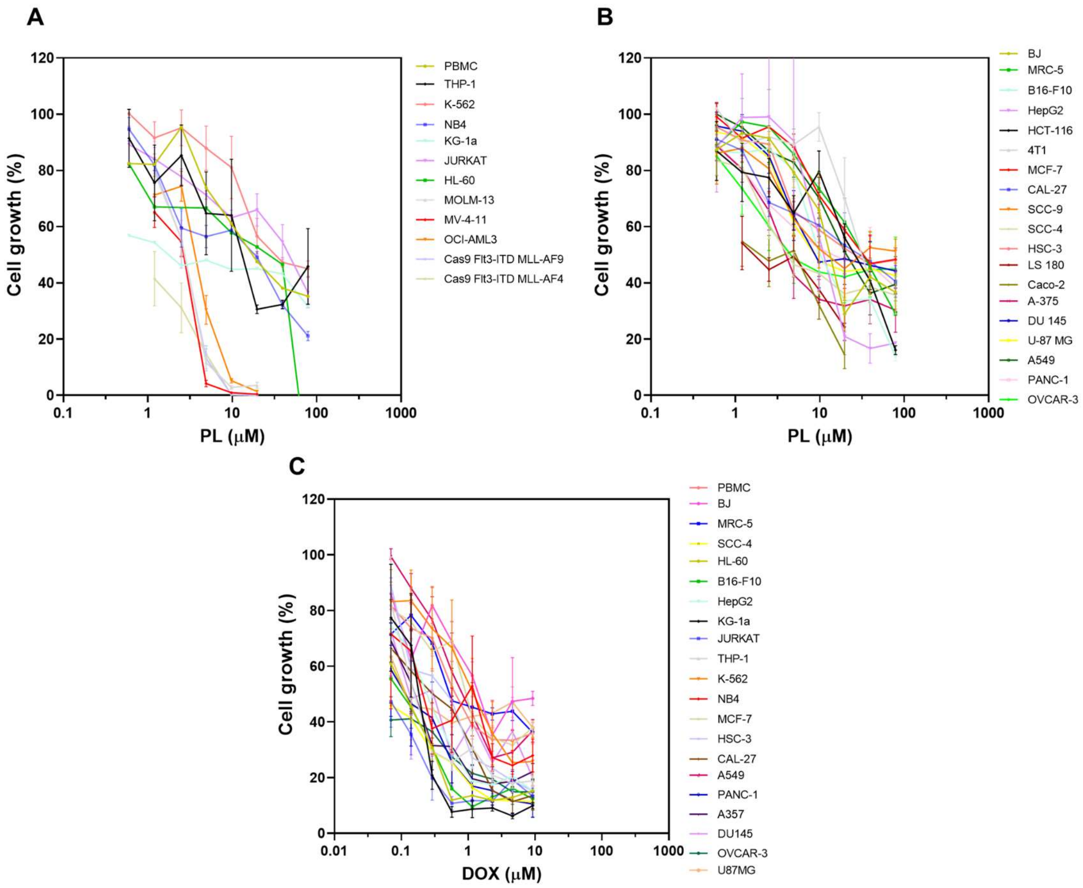

Figure S1. Cell growth curves obtained after 72 h of incubation with PL. (**A**) Growth curves of leukaemia cells and PBMCs and (**B**) cancer cell lines derived from solid tumours and the noncancerous cell lines BJ and MRC-5 after PL treatment. (**C**) Growth curves of cancer cells and noncancerous cells after 72 h of incubation with the positive control doxorubicin. These data were obtained from three independent experiments performed in duplicate and measured either by the Alamar blue assay or trypan blue assay after 72 h of incubation.

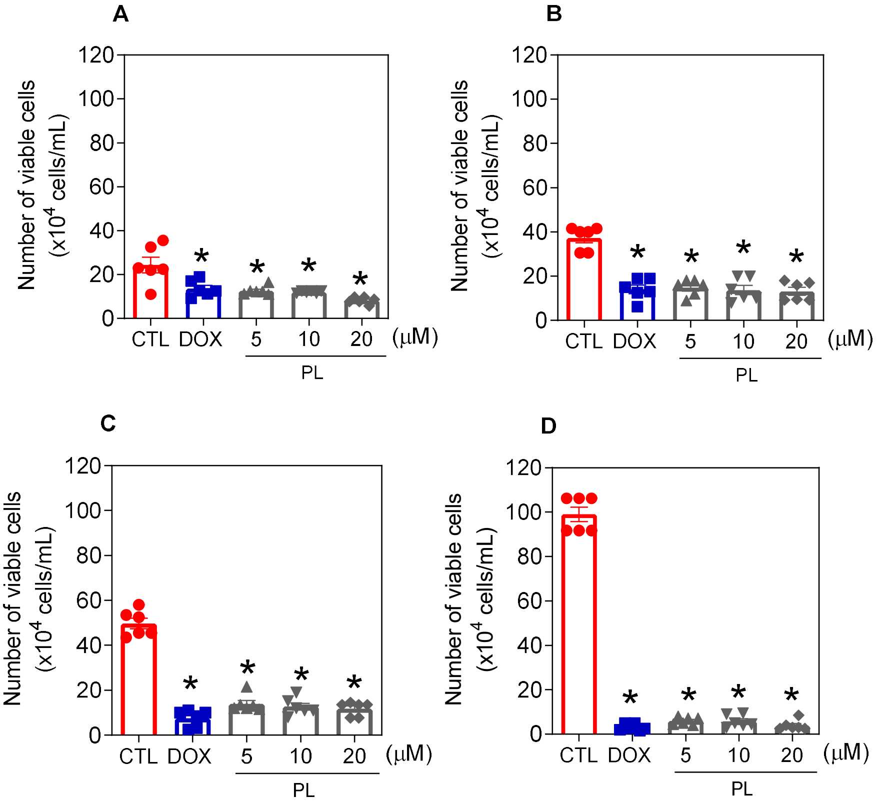

Figure S2. Trypan blue exclusion assay after (**A**) 12, (**B**) 24, (**C**) 48 and (**D**) 72 h of treatment with PL in KG-1a cells. Vehicle (0.2% DMSO) was used as a negative control (CTL), and doxorubicin (DOX, 1 µM) was used as a positive control. The data are expressed as the mean ± S.E.M. of three independent experiments carried out in duplicate. * $p < 0.05$ compared with CTL by one-way ANOVA followed by Dunnett's multiple comparisons test.

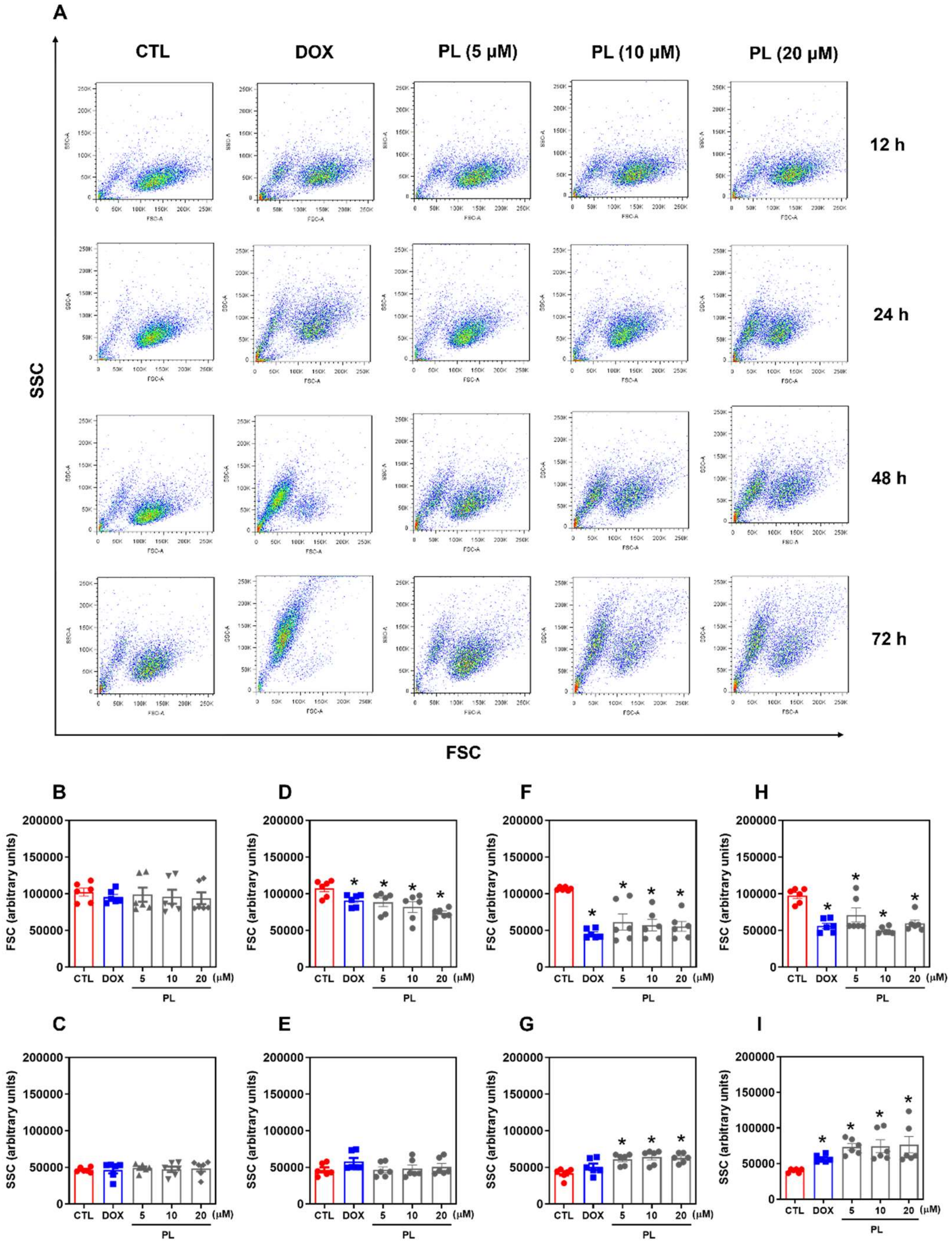

Figure S3. Effect of PL on the morphology of KG-1a cells, as determined by light-scattering features detected by flow cytometry after 12 (**A, B** and **C**), 24 (**A, D** and **E**), 48 (**A, F** and **G**) and 72 (**A, H** and **I**) h of incubation. The vehicle (0.2% DMSO) was used as a control (CTL), and doxorubicin (DOX, 1 μM) was used as a positive control. The data are shown as the mean ± S.E.M. of three independent experiments carried out in duplicate. * $P < 0.05$ compared to CTL by one-way ANOVA followed by Dunnett's multiple comparisons test.

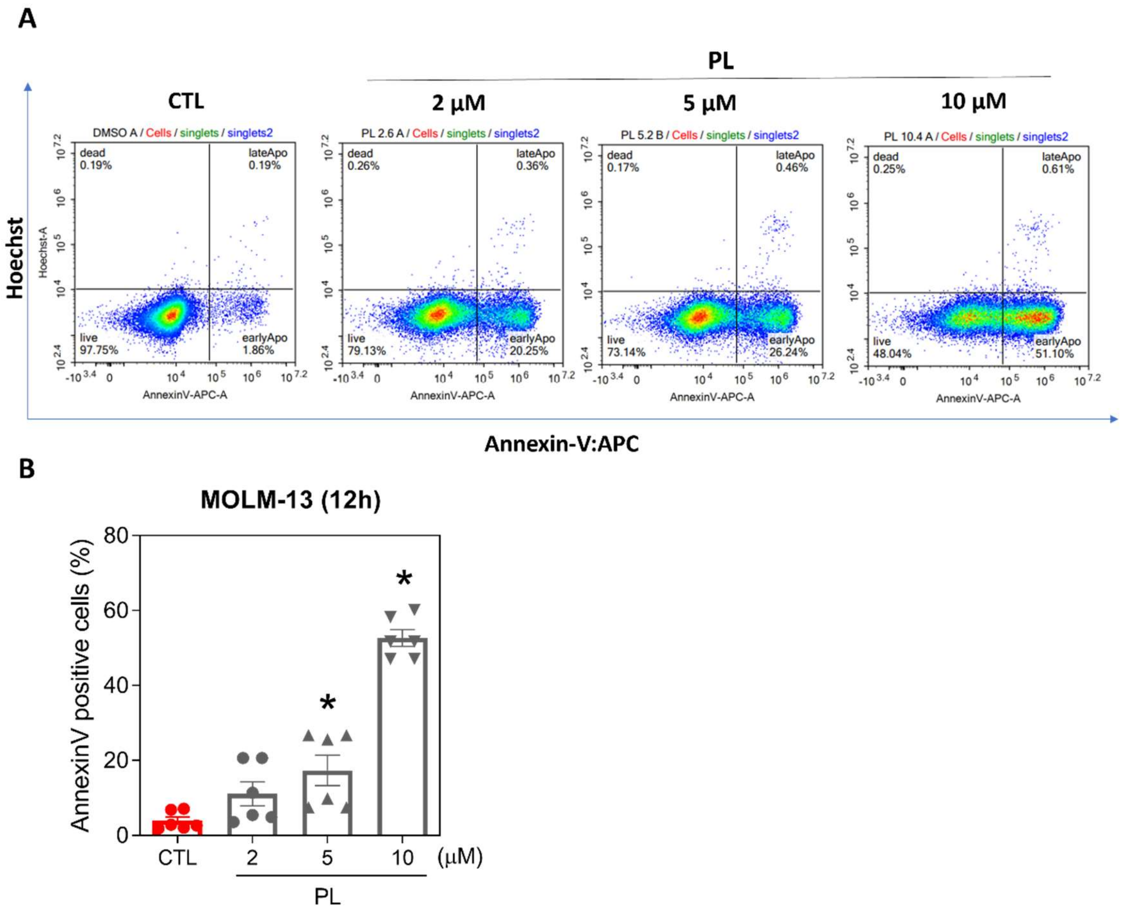

Figure S4. Apoptotic MOLM-13 cell death was induced by PL. (**A**) Representative flow cytometry dot plots. (**B**) Apoptosis quantification in MOLM-13 cells after 12 h of treatment with PL. The vehicle (0.2% DMSO) was used as a negative control (CTL). The data are shown as the mean ± S.E.M. of three independent experiments carried out in duplicate. * $p < 0.05$ compared with CTL by one-way ANOVA followed by Dunnett's multiple comparisons test.

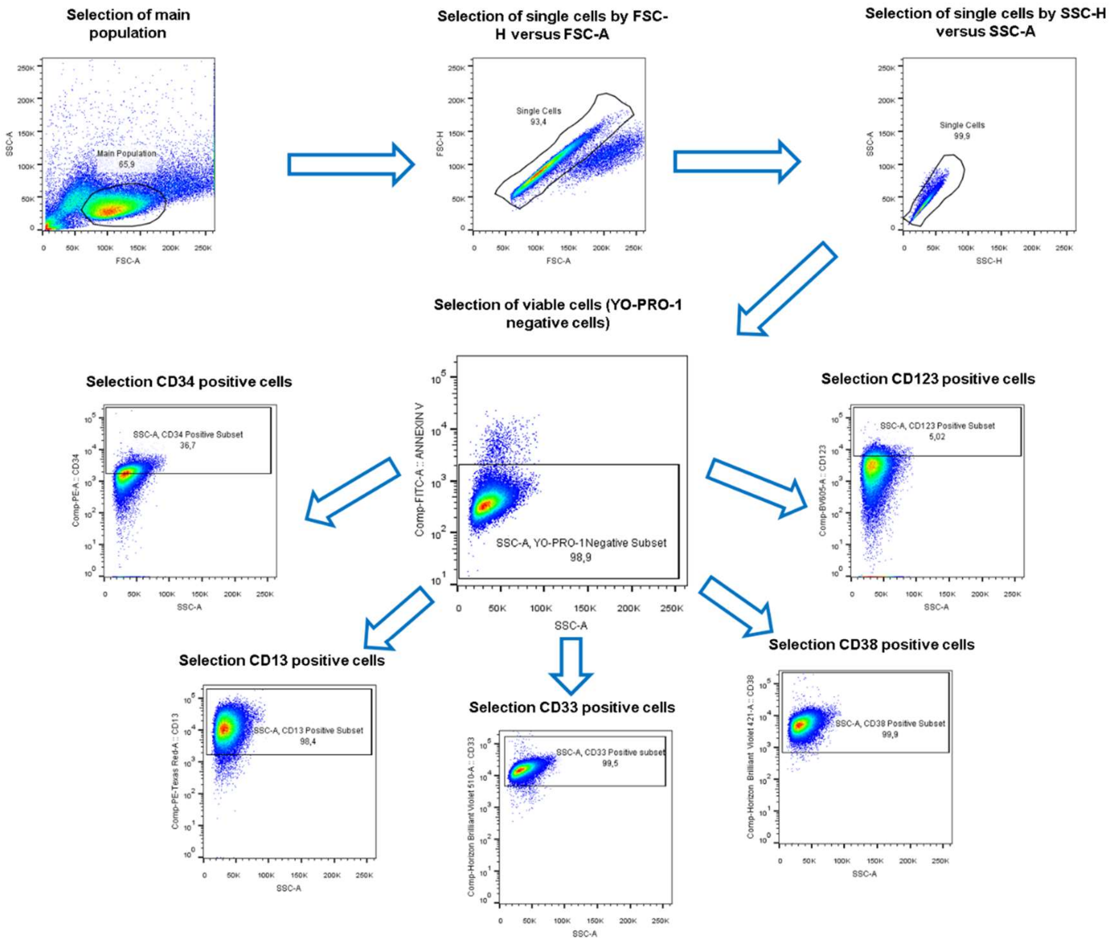

Figure S5. A representative strategy for immunophenotyping of KG1a cells used in flow cytometry analysis. PE mouse anti-human CD34, BV421 mouse anti-human CD38, BV605 mouse anti-human CD123, PE-CF594 mouse anti-human CD13 and BV510 mouse anti-human CD33 antibodies were used. PE mouse IgG1, a κ isotype control, was used as an isotype control, and annexin V-FITC (BD Biosciences) or YO-PRO-1 was used to select viable cells.

Table S1. Cytotoxicity of PL.

Cells	IC$_{50}$ and 95% CI (µM)	
	**PL**	**DOX**
Cancer cells		
Cas9 Flt3-ITD MLL-AF4	0.96<br>0.60 - 1.54	N.D.
MV-4-11	2.05<br>1.76 - 2.40	N.D.
Cas9 Flt3-ITD MLL-AF9	2.55<br>2.15 – 3.02	N.D.
MOLM-13	2.58<br>2.32 - 2.87	N.D.
KG-1a	2.83<br>0.5 – 14.7	0.17<br>0.13 – 0.22
OCI-AML-3	3.21<br>2.66 – 3.89	N.D.
HL-60	8.25<br>2.42 – 28.1	0.34<br>0.23 – 0.48
NB4	23.66<br>11.86 – 47.21	0.46<br>0.14 – 1.58
THP-1	23.87<br>7.88 – 72.28	0.25<br>0.016 – 0.44
K-562	41.29	1.53

	27.91 – 61.09	0.85 – 2.77
Jurkat	41.83	0.01
	26.66 – 65.61	0.01 – 0.54
Caco-2	2.57	N.D.
	1.36 - 4.88	
LS 180	2.23	N.D.
	1.03 – 4.81	
HSC-3	4.69	0.53
	3.32 – 6.61	0.39 – 0.73
A-375	6.8	0.16
	4.50 – 10.30	0.08 - 0.30
SCC-4	8.90	2.1
	5.74 – 13.8	1.7 – 2.6
CAL27	10.88	0.34
	7.31 – 16.20	1.65 – 0.69
HepG2	11.96	0.32
	6.20 – 23.09	0.18 -0.55
OVCAR-3	12.95	0.03
	5.35 – 31.32	0.01 - 0.08
B16-F10	14.50	0.15
	10.22 – 20.53	0.07 – 0.18
PANC-1	19.44	0.12
	6.57 – 57.53	0.05 – 0.26
HCT116	19.61	0.09
	11.59 – 33.16	0.05 – 0.16

U87-MG	20.82	0.17
	11.5 – 37.8	0.01 – 1.64
DU 145	24.34	0.24
	14.20 – 41.80	0.07 – 0.85
A549	27.34	1.17
	19.53 – 38.25	0.74 – 1.83
4T1	33.11	1.62
	23.18 – 47.34	1.10 – 2.35
MCF-7	38.99	1.51
	26.12 – 58.20	0.96 – 2.38
SCC-9	42.04	1.01
	15.39 – 114.9	0.60 – 1.73
Noncancerous cells		
BJ	20.34	3.60
	11.95 – 34.62	1.15 – 11.22
PBMC	21.91	1.21
	12.01 – 39.98	0.73 – 1.99
MRC-5	34.19	1.65
	21.48 – 54.42	1.03 – 2.61

These data were calculated by nonlinear regression from three independent experiments carried out in duplicate. Doxorubicin (DOX) was used as a positive control. N.D. = not determined.

Table S2. The effect of PL on gene expression in KG-1a cells.

Function/Assay ID	Gene Symbol	Gene Name	RQ	
			CTL	PL
**NFkB pathway**				
Hs00765730_m1	NFKB1	nuclear factor kappa B subunit 1	1.00	0.34
Hs00174517_m1	NFKB2	nuclear factor kappa B subunit 2	1.00	1.17
Hs00153283_m1	NFKBIA	NFKB inhibitor alpha	1.00	2.60
Hs00182115_m1	NFKBIB	NFKB inhibitor beta	1.00	2.19
Hs00153294_m1	RELA	RELA proto-oncogene, NF-kB subunit	1.00	2.64
Hs00232399_m1	RELB	RELB proto-oncogene, NF-kB subunit	1.00	3.86
**WNT/β-catenin pathway**				
Hs00181051_m1	APC	APC, WNT signalling pathway regulator	1.00	2.67
Hs00793391_m1	CSNK1A1	casein kinase 1 alpha 1	1.00	4.60
Hs00170025_m1	CTNNB1	catenin beta 1	1.00	2.88
Hs00275656_m1	GSK3B	glycogen synthase kinase 3 beta	1.00	4.08
Hs00228741_m1	WNT10A	Wnt family member 10A	n.d.	n.d.
Hs00559664_m1	WNT10B	Wnt family member 10B	1.00	n.d.
Hs00257131_m1	WNT2B	Wnt family member 2B	n.d.	n.d.
Hs00362452_m1	WNT6	Wnt family member 6	n.d.	n.d.
**Hedgehog pathway**				

Hs00368306_m1	DHH	desert hedgehog	n.d.	n.d.
Hs00171790_m1	GLI1	GLI family zinc finger 1	1.00	1.30
Hs00257977_m1	GLI2	GLI family zinc finger 2	n.d.	n.d.
Hs00181117_m1	PTCH1	patched 1	1.00	0.46
Hs00179843_m1	SHH	sonic hedgehog	n.d.	n.d.
Hs00170665_m1	SMO	smoothened, frizzled class receptor	n.d.	n.d.
Hs00171981_m1	SUFU	SUFU negative regulator of hedgehog signalling	1.00	2.66
**NOTCH pathway**				
Hs00194509_m1	DLL1	delta like canonical Notch ligand 1	1.00	9.59
Hs01085096_m1	DLL3	delta like canonical Notch ligand 3	1.00	21.01
Hs00164982_m1	JAG1	jagged 1	1.00	1.55
Hs00171432_m1	JAG2	jagged 2	1.00	5.14
Hs01062014_m1	NOTCH1	notch 1	1.00	0.45
Hs01050702_m1	NOTCH2	notch 2	1.00	0.99
**EGFR pathway**				
Hs01099999_m1	EGF	epidermal growth factor	1.00	30.45
Hs01076078_m1	EGFR	epidermal growth factor receptor	n.d.	n.d.
Hs00364282_m1	KRAS	KRAS proto-oncogene, GTPase	1.00	2.00
Hs01046830_m1	MAPK1	mitogen-activated protein kinase 1	1.00	2.66

Hs00234119_m1	RAF1	Raf-1 proto-oncogene, serine/threonine kinase	1.00	1.77
Hs00269660_s1	RHOB	ras homolog family member B	n.d.	n.d.
**JAK/STAT pathway**				
Hs01026983_m1	JAK1	Janus kinase 1	1.00	2.16
Hs01078136_m1	JAK2	Janus kinase 2	1.00	2.12
Hs00169663_m1	JAK3	Janus kinase 3	n.d.	n.d.
Hs01013989_m1	STAT1	signal transducer and activator of transcription 1	1.00	1.33
Hs00374280_m1	STAT3	signal transducer and activator of transcription 3	1.00	4.28
Hs00273500_m1	STAT5B	signal transducer and activator of transcription 5B	1.00	0.47
Hs00598625_m1	STAT6	signal transducer and activator of transcription 6	1.00	0.39
**PI3K/AKT/MTOR pathway**				
Hs00178289_m1	AKT1	AKT serine/threonine kinase 1	1.00	0.57
Hs01086102_m1	AKT2	AKT serine/threonine kinase 2	1.00	0.94
Hs00234508_m1	MTOR	mechanistic target of rapamycin	1.00	1.52
Hs00904054_m1	PIK3C2A	phosphatidylinositol-4-phosphate 3-kinase catalytic subunit type 2 alpha	1.00	5.88

Hs00176908_m1	PIK3C3	phosphatidylinositol 3-kinase catalytic subunit type 3	1.00	3.46
Hs00907957_m1	PIK3CA	phosphatidylinositol-4,5-bisphosphate 3-kinase catalytic subunit alpha	1.00	3.54
Hs02621230_s1	PTEN	phosphatase and tensin homolog	1.00	0.51
**TGF-beta/SMAD pathway**				
Hs01054576_m1	FOXO1	forkhead box O1	1.00	4.51
Hs00183425_m1	SMAD2	SMAD family member 2	1.00	3.31
Hs00929647_m1	SMAD4	SMAD family member 4	1.00	2.81
Hs00178696_m1	SMAD7	SMAD family member 7	1.00	0.54
Hs00998133_m1	TGFB1	transforming growth factor beta 1	1.00	0.75
Hs00234244_m1	TGFB2	transforming growth factor beta 2	1.00	7.89
Hs01086000_m1	TGFB3	transforming growth factor beta 3	1.00	3.97
**PPAR pathway**				
Hs00947536_m1	PPARA	peroxisome proliferator activated receptor alpha	1.00	1.74
Hs04187066_g1	PPARD	peroxisome proliferator activated receptor delta	1.00	2.50
Hs01115513_m1	PPARG	peroxisome proliferator activated receptor gamma	1.00	2.39
Hs01016719_m1	PPARGC1A	PPARG coactivator 1 alpha	n.d.	n.d.
Hs00991677_m1	PPARGC1B	PPARG coactivator 1 beta	1.00	0.19

**Oxidative stress**				
Hs00943350_g1	GSTP1	glutathione S-transferase pi 1	1.00	0.97
Hs00178247_m1	OXSR1	oxidative stress responsive 1	1.00	1.91
Hs00167309_m1	SOD2	superoxide dismutase 2, mitochondrial	1.00	2.98
Hs01555214_g1	TXN	thioredoxin	1.00	2.47
Hs00917067_m1	TXNRD1	thioredoxin reductase 1	1.00	21.94
**Apoptosis**				
Hs00559441_m1	APAF1	apoptotic peptidase activating factor 1	1.00	2.55
Hs00188930_m1	BAD	BCL2 associated agonist of cell death	1.00	0.97
Hs00180269_m1	BAX	BCL2 associated X, apoptosis regulator	1.00	0.70
Hs99999018_m1	BCL2	BCL2, apoptosis regulator	1.00	0.11
Hs00609632_m1	BID	BH3 interacting domain death agonist	1.00	0.77
Hs00234387_m1	CASP3	caspase 3	1.00	7.36
Hs00169152_m1	CASP7	caspase 7	1.00	1.04
Hs00219876_m1	DIABLO	diablo IAP-binding mitochondrial protein	1.00	2.66
Hs00538709_m1	FADD	Fas associated via death domain	1.00	1.71
Hs00531110_m1	FAS	Fas cell surface death receptor	1.00	1.13
Hs00242302_m1	PARP1	poly(ADP-ribose) polymerase 1	1.00	0.41

**Autophagy**				
Hs01047860_g1	ATG12	autophagy related 12	1.00	3.20
Hs00223937_m1	ATG3	autophagy related 3	1.00	0.90
Hs00169468_m1	ATG5	autophagy related 5	1.00	3.94
Hs00186838_m1	BECN1	beclin 1	1.00	1.18
Hs01076567_g1	MAP1LC3A	microtubule associated protein 1 light chain 3 alpha	1.00	1.93
Hs00797944_s1	MAP1LC3B	microtubule associated protein 1 light chain 3 beta	1.00	44.71
**Necroptosis**				
Hs00169407_m1	RIPK1	receptor interacting serine/threonine kinase 1	1.00	4.82
Hs01572686_m1	RIPK2	receptor interacting serine/threonine kinase 2	1.00	0.69
Hs01011177_g1	RIPK3	receptor interacting serine/threonine kinase 3	1.00	0.38
**Epithelial–mesenchymal transition**				
Hs00170423_m1	CDH1	cadherin 1	n.d.	n.d.
Hs00195591_m1	SNAI1	snail family transcriptional repressor 1	1.00	12.69
Hs00950344_m1	SNAI2	snail family transcriptional repressor 2	n.d.	n.d.
Hs01018996_m1	SNAI3	snail family transcriptional repressor 3	1.00	0.11

Hs00361186_m1	TWIST1	twist family bHLH transcription factor 1	1.00	0.10
Hs00185584_m1	VIM	vimentin	1.00	1.95
Hs00232783_m1	ZEB1	zinc finger E-box binding homeobox 1	1.00	5.02
Hs00207691_m1	ZEB2	zinc finger E-box binding homeobox 2	1.00	2.50

KG-1a cells were treated with 20 μM PL for 12 h. The negative control (CTL) was treated with the vehicle (0.2% DMSO) used for diluting the compound tested. After treatment, total RNA was isolated and reverse transcribed. Gene expression was detected using a TaqMan® array plate 96 plus fast (#4413256). The GUSB, HPRT1 and GAPDH genes were used as endogenous genes for normalization. The values represent the relative quantitation (RQ) compared with the calibrator (cells treated with the negative control). The genes were considered to be upregulated if RQ ≥ 2 and downregulated if RQ ≤ 0.5. N.d. Not determined.

Table S3. List of cells used.

Cells	Histological type	Species	Source[a,b,c,d,e]
Cancer cell lines			
MV-4-11	acute myelogenous leukemia	human	HL
MOLM-13	acute myelogenous leukemia	human	HL
KG-1a	acute myelogenous leukemia	human	ATCC
OCI-AML-3	acute myelogenous leukemia	human	HL
NB4	acute promyelocytic leukemia	human	ATCC
THP-1	monocytic leukemia	human	ATCC
JURKAT	T-cell lymphoid leukemia	human	ATCC
K-562	chronic myelogenous leukemia	human	ATCC
HL-60	acute promyelocytic leukemia	human	ATCC
Caco-2	colorectal adenocarcinoma	human	ABL
LS 180	colorectal adecnocarcinoma	human	ABL
HCT116	colorectal carcinoma	human	ATCC
HepG2	hepatocellular carcinoma	human	ATCC
KG-1a	acute myeloid leukemia	human	ATCC
MCF-7	breast adenocarcinoma	human	ATCC
4T1	breast carcinoma	mouse	ATCC
HSC-3	oral squamous cell carcinoma	human	ATCC
CAL 27	oral squamous cell carcinoma	human	ATCC
SCC4	oral squamous cell carcinoma	human	ATCC
SCC-9	oral squamous cell carcinoma	human	ATCC

A549	lung adenocarcinoma	human	BCRJ
PANC-1	pancreas ductal adenocarcinoma	human	BCRJ
OVCAR-3	ovarian carcinoma	human	BCRJ
DU 145	prostate carcinoma	human	BCRJ
U-87 MG	glioblastoma	human	BCRJ
A-375	melanoma	human	BCRJ
B16-F10	melanoma	mouse	ATCC
Noncancer cell lines			
MRC-5	lung fibroblast	human	ATCC
BJ	foreskin fibroblast	human	ATCC
Mutant and its parental cell lines			
BAD KO SV40 MEF	immortalized mouse embryonic fibroblasts with the BAD gene knocked out	mouse	ATCC
WT SV40 MEF	wild-type immortalized embryonic fibroblasts	mouse	ATCC
Primary cells			
Cas9 Flt3-TD MLL-AF4	bone marrow	mouse	primary cell culture
Cas9 Flt3-ITD MLL-AF9	bone marrow	mouse	primary cell culture
C57BL/6	bone marrow	mouse	primary cell culture
PBMC	health peripheral blood mononuclear cells	human	primary cell culture

[a]ATCC denotes the American Type Culture Collection (USA), and BCRJ denotes the Rio de Janeiro Cell Bank (Brazil). [b]ABL are cells that were kindly donated by Annabelle Lewis from Brunel University London and were maintained in DMEM (Gibco®) supplemented with 10% FBS and 1% penicillin/streptomycin/amphotericin (Gibco®). [c]Primary mouse bone marrow cells from a Roas26-Cas9 Flt3-ITD mouse model were transformed in vitro through retroviral transduction with the MLL-AF9 or MLL-AF4 oncogene, and serial replating in colony-forming cell assays for leukaemia clone selection was performed as previously described [1]. Cas9 Flt3-ITD MLL-AF9 or MLL-AF4 cells were maintained in XVIVO-15 supplemented with 1% penicillin/streptomycin/amphotericin (Gibco®), 10 ng/mL murine interleukin 3 (mIL-3), 10 ng/mL murine interleukin 6 (mIL-6), and 20 ng/mL murine stem cell factor (mSCF). [d]Primary cell culture of PBMCs was obtained from peripheral blood from healthy donors by a standard Ficoll density protocol. Then, the PBMCs were resuspended in RPMI 1640 or DMEM-F12 supplemented with 20% FBS and 1% antibiotics. The cells were plated at 5×10^5 cells/well. Concanavalin A (10 µg/mL; Sigma–Aldrich), which was added at the beginning of the culture, was used as a mitogen to trigger cell division in T lymphocytes. The Research Ethics Committee of the Oswaldo Cruz Foundation (Salvador, Bahia, Brazil) approved the protocol (CAAE 16220713.2.0000.0040). [e]MV-4-11, MOLM-13 and OCI-AML3 cells were donated by the Huntly Laboratory or obtained from the Sanger Institute. These findings were verified against the basis of cell surface marker data in the DSMZ database.

1. Tzelepis K, Koike-Yusa H, De Braekeleer E, Li Y, Metzakopian E, Dovey OM, et al. A CRISPR dropout screen identifies genetic vulnerabilities and therapeutic targets in acute myeloid leukemia. Cell Rep. 2016 Oct 18;17(4):1193-1205. doi: 10.1016/j.celrep.2016.09.079.

Table S4. Antibodies used.

Antibody	Fluorochrome	Reactivity	Clone	Catalog number	Manufacturer	Dilution
Active caspase-3	FITC	Human/ Mouse	C92-605	559341	BD Pharmingen™	1:20
Cleaved PARP (Asp214)	PE	Human	F21-852	552933	BD Pharmingen™	1:10
NFkB p65 (pS529)	AF488	Human	K108951 250	558421	BD Phosflow™	1:20
NFkB p65 (pS536)	FITC	Human	NFKBp65 S536-B7	MA5-37157	Thermo Scientific	1:20
NFkB p65	PE	Human	14G10A2 1	653004	BioLegend	1:20
CD13	PE-CF594	Human	WM15	562491	BD Horizon™	1:400
CD33	BV510	Human	WM53	563257	BD Horizon™	1:400
CD123	BV605	Human	7G3	564197	BD Horizon™	1:100
CD34	PE	Human	8G12	348057	BD™	1:10
CD38	BV421	Human	HIT2	562444	BD Horizon™	1:400

IgG1, κ Isotype Control	PE	-	MOPC-21	556650	BD Pharmingen™	1:100
CD45 (hCD45)	PE	Human	2D1	368509	BioLegend	1:1000
CD45 (mCD45)	FITC	Mouse	30-F11	103107	BioLegend	1:1000
